# Ultrapermeable 2D-channeled graphene-wrapped zeolite molecular sieving membranes for hydrogen separation

**DOI:** 10.1126/sciadv.abl3521

**Published:** 2022-05-18

**Authors:** Radovan Kukobat, Motomu Sakai, Hideki Tanaka, Hayato Otsuka, Fernando Vallejos-Burgos, Christian Lastoskie, Masahiko Matsukata, Yukichi Sasaki, Kaname Yoshida, Takuya Hayashi, Katsumi Kaneko

**Affiliations:** 1 Research Initiative for Supra-Materials, Shinshu University, 4-17-1 Wakasato, Nagano 380-8553, Japan.; 2 Center for Biomedical Research, Faculty of Medicine, University of Banja Luka, Save Mrkalja 14, Banja Luka 78000, Bosnia and Herzegovina.; 3 Research Organization for Nano and Life Innovation, Waseda University, 513 Waseda-Tsurumaki-cho, Shinjuku-ku, Tokyo 162-0041, Japan.; 4 Morgan Advanced Materials, Carbon Science Centre of Excellence, 310 Innovation Blvd., Suite 250, State College, PA 16803, USA.; 5 Department of Civil and Environmental Engineering, University of Michigan, 1351 Beal Avenue, Ann Arbor, MI 48109-2125, USA.; 6 Department of Applied Chemistry, Waseda University, 513 Wasedatsurumaki-cho, Shinjuku-ku, Tokyo 162-0041, Japan.; 7 Advanced Research Institute for Science and Engineering, Waseda University, 513 Wasedatsurumaki-cho, Shinjuku-ku, Tokyo 162-0041, Japan.; 8 Nanostructures Research Laboratory, Japan Fine Ceramics Center, 2-4-1 Mutsuno, Atsuta-ku, Nagoya 456-8587, Japan.; 9 Department of Water Environment and Civil Engineering, Shinshu University, 4-17-1 Wakasato, Nagano 380-8553, Japan.

## Abstract

The efficient separation of hydrogen from methane and light hydrocarbons for clean energy applications remains a technical challenge in membrane science. To address this issue, we prepared a graphene-wrapped MFI (G-MFI) molecular-sieving membrane for the ultrafast separation of hydrogen from methane at a permeability reaching 5.8 × 10^6^ barrers at a single gas selectivity of 245 and a mixed gas selectivity of 50. Our results set an upper bound for hydrogen separation. Efficient molecular sieving comes from the subnanoscale interfacial space between graphene and zeolite crystal faces according to molecular dynamic simulations. The hierarchical pore structure of the G-MFI membrane enabled rapid permeability, indicating a promising route for the ultrafast separation of hydrogen/methane and carbon dioxide/methane in view of energy-efficient industrial gas separation.

## Teaser

Graphene-wrapped MFI membranes selectively separate H_2_ from CH_4_ with two orders higher H_2_ permeability than the present ones.

## INTRODUCTION

H_2_ is a notable industrial target for clean energy generation and intensive CO_2_ reduction (*1*, *2*), and it is mainly produced through steam reforming of natural gas (*3*). The energy-saving recovery of H_2_ from refinery streams containing H_2_, CH_4_, and light hydrocarbons is ultimately aimed at consistently reducing CO_2_ emissions (*2*, *4*). The recovery of H_2_ from refinery gases via membrane separation is more favorable than distillation with regard to energy consumption and reduction of CO_2_ emissions (*5*–*7*). The steam reforming process of natural gas is performed at a high temperature of approximately 1000 K (*3*); thus, thermally stable membranes are preferred for an energy-saving process.

Zeolite membranes are thermally stable and robust and are promising candidates for application in the steam reforming process. However, zeolites with small pores pose a challenge because their pore sizes are larger than the molecular size of CH_4_. MFI zeolites with a uniform pore size of 0.55 nm (*8*) are suitable candidates for membrane fabrication and have been extensively studied for high-performance separation processes. Recent study of membranes has reported progress in MFI zeolite membranes in which crack-free MFI membranes were prepared using exfoliated MFI nanosheets (*9*). However, the pore sizes of the MFI membranes are larger than the molecular sizes of the target H_2_ and CH_4_; therefore, the selectivity of H_2_/CH_4_ was limited to 25 (*10*). Although the crack issue in zeolite membranes is addressed by the development of a layered zeolite membrane route, the establishment of an energy-saving separation technology requires a new type of thermally stable zeolite-based membrane that enables the rapid and selective separation of H_2_ from CH_4_ or other gases.

Therefore, the development of outstanding zeolite-based membranes for the construction of H_2_-assisted green technology is an active challenge (*11*). Internal surface modification of MFI zeolite channels reduces the effective size of the channels, achieving a H_2_/CH_4_ selectivity of 74 with H_2_ permeance of 4.9 × 10^−8^ mol m^−2^ s^−1^ Pa^−1^ at 300 K (*12*) and a H_2_/CO_2_ selectivity of 25 with H_2_ permeance of 1.28 × 10^−7^ mol m^−2^ s^−1^ Pa^−1^ at 723 K (*13*). Mixed matrix membranes (MMMs) consisting of zeolites and other porous fillers have been explored as high-performance membranes (*14*, *15*). A hollow silicalite-1 (MFI) MMM exhibited a H_2_/CH_4_ selectivity of 180 at a permeance of ~10^−8^ mol m^−2^ s^−1^ Pa^−1^ at 300 K (*16*). Nevertheless, the performance of MMMs is still inadequate, and the development of membranes with high permeance and concurrent high selectivity is crucial. In comparison to polymer-based membranes, inorganic porous membranes can achieve the aforementioned properties (*17*). This study focused on the development of zeolite-based membranes as a class of inorganic membranes.

A promising solution consists of using a membrane made of small zeolite crystals wrapped with colloidal graphene sheets bearing nanoscale holes (nanowindows) (*18*, *19*). Target gases permeate through the nanowindows (*20*) and access the interfacial spaces between graphene and zeolite crystal surfaces. The graphene-graphene attractive interactions are the strongest per weight because of the densely packed carbon atoms in the graphene structure (*21*). Consequently, the graphene-wrapped zeolite particles adhere to each other through face-to-face and/or edge-shared contacts via van der Waals interactions (*22*), providing a crack-free membrane through a simple compression method. The zeolite crystal faces of uneven groove structures provide a graphene-zeolite interfacial space that can sieve gases depending on their molecular sizes.

If the nanowindows are large enough to host the target H_2_ molecules and the interfacial space between graphene and the zeolite surface fits only H_2_ molecules, then H_2_ can selectively permeate through the interfacial spaces. The permeance through these graphene-zeolite interfacial channels is larger than that of the intrinsic three-dimensional (3D) interconnected MFI channels. Our experimental observations confirmed that the MFI crack-free membrane (*23*, *24*) exhibited a H_2_ permeance of 3.6 × 10^−7^ mol m^−2^ s^−1^ Pa^−1^ and a H_2_/CH_4_ selectivity of 1.41, whereas the graphene-MFI interfacial space channeled membrane exhibited a permeance of 1.3 × 10^−5^ mol m^−2^ s^−1^ Pa^−1^ and a H_2_/CH_4_ selectivity of 245. Thus, the process of wrapping can create subnanoscale graphene-zeolite interfacial channels that are smaller than intrinsic zeolite channels (that is, 0.55 nm for MFI) and beneficial for energy-saving ultrafast separation. Introducing a hierarchical pore structure in the membrane by controlling the aggregation structure of the graphene-wrapped zeolite particles enhanced the permeance of the membrane. For smaller zeolite crystal sizes, shorter paths were formed; thus, the permeance in the interfacial spaces further increased. The apparent thickness of the graphene-wrapped MFI (G-MFI) membranes reached hundreds of micrometers. However, the G-MFI membrane with a large intergranular porosity enabled the high permeance; the effective permeation length of the graphene-MFI interface, which determines the permeation rate, should be extremely small in comparison with the apparent thickness of the G-MFI membrane, as suggested elsewhere (*25*, *26*). Furthermore, the graphene-wrapped zeolite membranes provided sufficient thermal stability because the colloidal graphene produced from graphene oxides (GOs) was stable up to 600 K (*27*). In this study, MFI zeolites were used owing to the intrinsic surface groove structure (*28*) that turned into the interfacial channels available for molecular sieving upon wrapping. The ultrafast and highly selective permeation of H_2_ through a G-MFI zeolite membrane was reported.

## RESULTS

### G-MFI membrane and separation efficiency

G-MFI crystals were prepared via a colloidal method on the basis of tuning the surface charges of GO and MFI crystals with NH_4_Cl (fig. S1, A to C). The GO-wrapped MFI crystals were thermally treated to reduce GO to graphene in an Ar atmosphere and were thermally stable, as confirmed by thermogravimetric analysis and Raman spectroscopic analyses (fig. S1, D to F). The thermogravimetric curve shows distinctive weight losses at 440 and 746 K owing to the thermal reduction of GO and burning of the reduced graphene. Raman spectroscopy indicates a D/G intensity ratio against temperature almost constant up to 623 K, providing that the structure of graphene on MFI is not altered upon thermal treatment in air, thus denoting the thermal stability of the G-MFI membrane. The membranes (0.6 cm by 0.6 cm) were prepared by compression of crystalline and spherical G-MFI powders at 550 MPa (fig. S1, G to J).

**Fig. 1.**
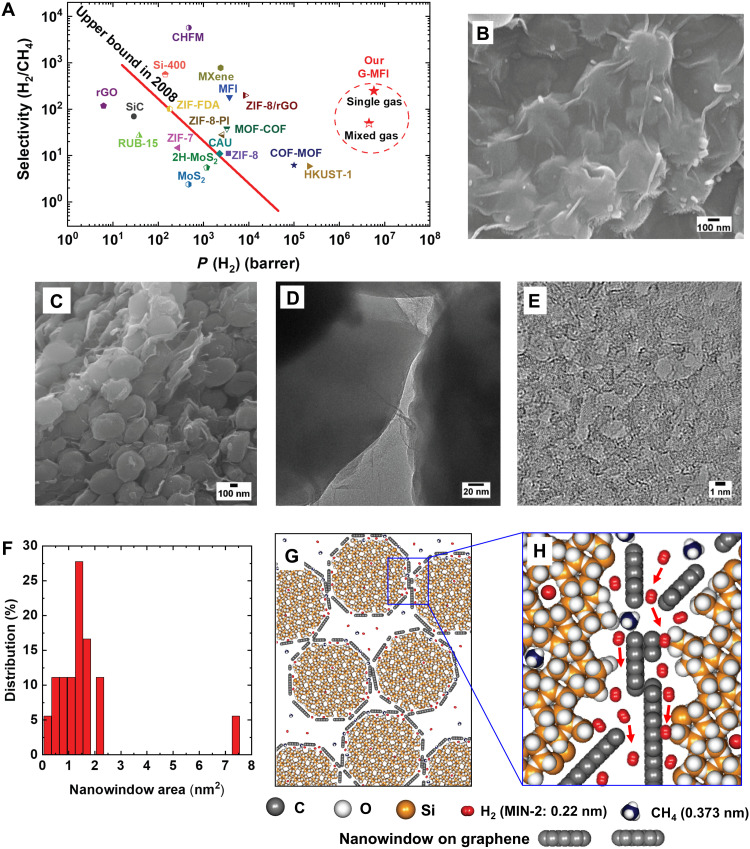
H_2_/CH_4_ separation efficiency achieved with G-MFI. (**A**) Robeson plot for H_2_/CH_4_ separation for single gas separation. For G-MFI membranes, the selectivity of both single and mixed gas is indicated. The red line refers to the upper bound proposed by Robeson using polymeric membranes (*44*). Robeson plot details are listed in table S1. (**B**) G-MFI SEM image. (**C**) Fractured G-MFI SEM cross-sectional image. (**D**) TEM image highlighting the contact between two MFI crystals wrapped with graphene. (**E**) TEM image of nanowindows in graphene. (**F**) Nanowindow size distribution histogram. (**G**) Edge share model structure of G-MFI membrane, depicting intergranular voids; the (010) crystallographic face of MFI is shown. (**H**) Simplified interfacial model showing the cross-sectional view of the graphene and MFI crystal face of the G-MFI membrane. The nanowindows in the single graphene layer are expressed by blanks in the graphene layer, although a graphene layer continuously covers the zeolite crystal. TEM images show a few graphene layers with nanowindows covering an MFI zeolite crystal in the real G-MFI (fig. S4). Few-layer wrapping can be approximated with monolayer wrapping because the gas permeance between the layers is negligible.

As shown in Fig. 1A, the G-MFI membranes exhibit an outstanding H_2_ permeability compared to metalic-organic frameworks (MOFs) and other zeolitic membranes, ascribable to the highly microporous and mesoporous structure. The graphene layers wrapped the MFI crystals (Fig. 1B), enabling the facile production of a crack-free MFI membrane, as confirmed by scanning electron microscopy (SEM) and permeation experiments. Furthermore, the SEM image of the G-MFI membranes surface shows the presence of intergranular mesoscale pores in the crystal assemblies, produced by freeze-drying. These pores are also visible in the cross-sectional view of the fractured G-MFI membranes (Fig. 1C). The cross-sectional SEM image shows a sheet-like structure, consisting of mutually combined G-MFI particles. The sheet-like structures were firmly stacked with each other in the thickness direction. The G-MFI particles were slightly dispatched from the stacked layer structure, because of fracturing of the G-MFI membrane. Therefore, the cross-sectional SEM image confirms a membrane film formation consisting of continuous graphene-wrapped zeolite particles.

The G-MFI membrane was further investigated using transmission electron microscopy (TEM) (Fig. 1, D and E). MFI has a highly crystalline structure, with elliptically shaped micropores (0.55 nm), which are visible at the (010) crystal faces (fig. S2). Partial edge contact between the G-MFI crystals was observed using TEM (Fig. 1, C and E). The graphene layers allowed adherence among the MFI crystals, suppressing intergranular crack formation. Moreover, the TEM images show the presence of nanowindows in the graphene used to wrap the MFI crystals (Fig. 1E and fig. S3). The MFI particles were wrapped with a few layers of graphene (fig. S4, A to C). The hexagonal structure of G-MFI was clearly observed using inverse Fourier transform images (fig. S4, D to L). The nanowindows, characterized by a cross-sectional area in the range of 0.3 to 7.4 nm^2^ (Fig. 1F), are wider than the permeate molecules, whose molecular size ranges from 0.26 to 0.55 nm, suggesting a preferential path for gas diffusion.

Density measurement supported the G-MFI membrane formation, with edge-shared 3D porous structures (cross section shown in Fig. 1G). The G-MFI membrane void volume fraction (0.29) was determined from the ratio between the G-MFI membrane bulk density (1.25 g cm^−3^) and the MFI bulk density (1.76 g cm^−3^) (*8*). This value indicates the presence of intergranular voids, which enable very rapid permeation. The diffusion coefficient of H_2_ through the G-MFI membrane was determined to be 9.5 × 10^−2^ cm^2^ s^−1^ by approximation using solution diffusion transport (see Supplementary Text). The diffusion coefficient of CH_4_ was 1.1 × 10^−4^ cm^2^ s^−1^, and thus, the ratio of the H_2_ and CH_4_ diffusion coefficients gives a diffusivity selectivity of H_2_/CH_4_ as high as 850. On the contrary, the ratio of the solubility coefficients of 2.0 × 10^−4^ mol m^−3^ Pa^−1^ for H_2_ and 7.5 × 10^−4^ mol m^−3^ Pa^−1^ for CH_4_ yielded a solubility selectivity of 0.27. The solubility selectivity slightly affects the diffusivity selectivity, as suggested by eq. 2 in Supplementary Text, indicating that the predominant separation mechanism of G-MFI originates from the diffusion mechanism, not from the solubility mechanism. This is because G-MFI has definite geometrical nanoscale spaces between the graphene and MFI crystal faces, which can discern H_2_ from CH_4_. The diffusion of H_2_ through the G-MFI membrane is three orders of magnitude higher than that through liquid water (4.5 × 10^−5^ cm^2^ s^−1^) (*29*). This agrees with the high void fraction of 0.29 of the G-MFI membrane, as shown in the model (Fig. 1G). Figure 1H shows the key model structure of narrow graphene-zeolite interfacial channels for excellent selectivity, which was further confirmed by the molecular dynamics (MD) simulation described later.

### Adsorption

**Fig. 2.**
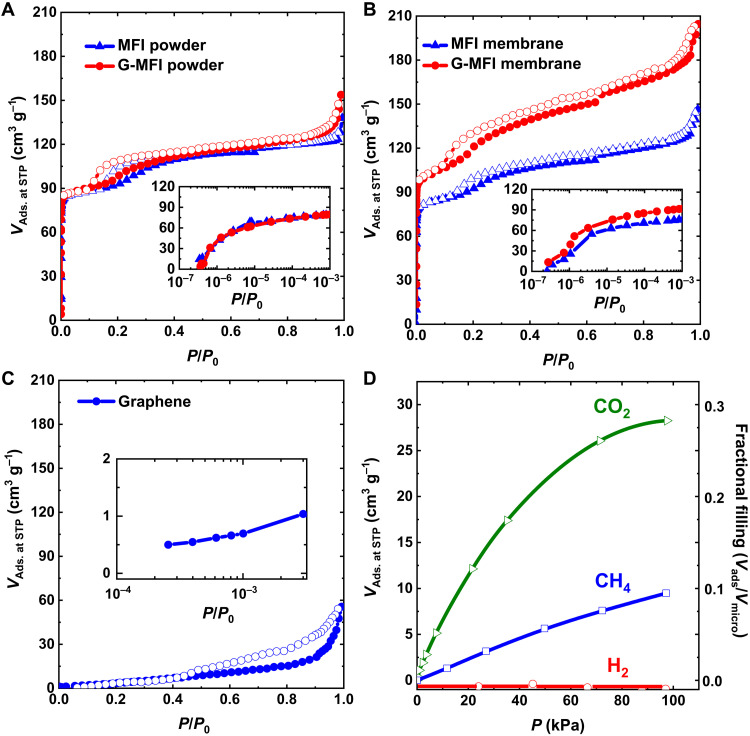
Porosity of G-MFI membranes as determined from N_2_ adsorption at 77 K. (**A**) N_2_ adsorption (Ads.) isotherms of G-MFI and MFI powder. Inset is the corresponding semilogarithmic plot. STP, standard temperature and pressure. (**B**) N_2_ adsorption isotherms of G-MFI and MFI membrane. Inset is the corresponding semilogarithmic plot. (**C**) N_2_ adsorption isotherm of compressed graphene. Inset is the corresponding semilogarithmic plot. (**D**) Adsorption isotherms and fractional fillings of H_2_, CO_2_, and CH_4_ plotted against pressure. The density of liquid H_2_ (0.0711 g cm^−3^ at 20 K and 10^5^ Pa), solid CO_2_ (1.566 g cm^−3^ at 193 K and 10^5^ Pa), and liquid CH_4_ (0.423 g cm^−3^ at 111 K and 10^5^ Pa) and micropore volume (table S3) of the G-MFI membrane were used to estimate the fractional fillings.

The porosity of both G-MFI powder and membrane were determined by N_2_ adsorption at 77 K (Fig. 2). The N_2_ adsorption isotherm shows a high uptake by micropores in the low *P*/*P*_0_ region. The hysteresis loop over the range 0.10 < *P*/*P*_0_ < 0.15 (Fig. 2A) arises from the in-pore phase transition of adsorbed N_2_ due to intensive confinement (*30*, *31*). A lower pressure shift is observed at the in-pore phase transition point. The reason for the shift is reasonably associated with a slight distortion of the MFI lattice during the graphene-wrapping and freeze-drying procedures, as indicated by the change in the full width at half maximum of the x-ray diffraction peaks (fig. S5A and table S2). The logarithmic plots of MFI and G-MFI powder (inset of Fig. 2A) show comparable N_2_ adsorption uptakes in the low-pressure region, suggesting that microporosity is preserved after graphene wrapping.

In contrast, the N_2_ adsorption isotherms of the G-MFI membrane at 77 K are remarkably different from those of the MFI membrane. In particular, the N_2_ adsorption amount is higher for G-MFI membrane (Fig. 2B). The enhanced uptake below *P*/*P*_0_ = 0.1 on the G-MFI membrane indicates the formation of very small micropores by wrapping procedure. The obtained micropores mainly originate from the interfacial space between graphene and MFI crystals, resulting in a slight increase in microporosity (table S3). The N_2_ adsorption isotherm related to the interfacial space was obtained by subtraction of the N_2_ adsorbed amount of G-MFI from that of the MFI membrane (fig. S6A). The pore size distribution obtained from the subtracted isotherm by quenched solid density functional theory for slit pores shows the presence of different pore sizes. Micropores are assigned to the interfacial space between graphene and MFI, whereas mesopores (3.8 to 6.0 nm) originate from the mutually aggregated structure of G-MFI particles, both in compression direction and plane direction (fig. S6B). Micropore and mesopore wide distribution suggests the presence of a hierarchical pore structure, which plays a prominent role in rapid permeation. Experimental micropore volume estimated from the subtracted G-MFI isotherm at 77 K, related to the adsorption at the interfacial space, was 0.02 cm^3^ g^−1^. This value indicates the presence of the narrow interfacial space available for the gas permeation. The theoretical graphene-MFI interfacial micropore volume was calculated by subtracting the MFI model pore volume from the G-MFI model pore volume. The obtained value of 0.03 ml g^−1^ is comparable with the experimental result, confirming the validity of the proposed G-MFI model (Fig. 1, G and H). The presence of intergranular micropores was also confirmed by the fact that small-angle x-ray scattering intensity of the G-MFI membrane is larger than that of MFI crystals (fig. S5, B and C). We estimated gyration radii (*R*_g_) from the linear region of Guinier plots (fig. S5C), considering the requirement (*QR*_g_ < 1.3) for fitting. The *R*_g_ of the G-MFI membrane is smaller than that of the MFI membrane, thereby suggesting a decrease in the size of micropores by graphene wrapping (table S4). The *R*_g_ of 0.48 nm suggests the presence of intergranular voids in the G-MFI membrane. The G-MFI particles were more mutually contacted than the compressed MFI membrane without graphene wrapping. Therefore, we assume that the graphene on the MFI crystal contributes to the crack-free membrane formation. In contrast, the strong graphene-graphene interaction suppressed the formation of micropores between adjacent G-MFI crystals, as shown by the adsorption amount below *P*/*P*_0_ = 0.1 (Fig. 2C).

The average micropore width of the interfacial space between graphene and the MFI crystal face was estimated to be ~0.40 nm, assuming the presence of slit-shaped micropores (*32*). However, N_2_ adsorption cannot evaluate the exact pore widths that are less than ~0.40 nm owing to the pore blocking effect. The width of the interfacial space, which induces the molecular sieving effect shown later, should be less than ~0.40 nm. The MD study further introduced provides reliable information about the interfacial space structure for molecular sieving.

Figure 2D reports the adsorption of the target gases adopted for separation, namely, H_2_, CH_4_, and CO_2_, on the G-MFI membranes. The supercritical H_2_ was not adsorbed. Supercritical CH_4_ interacts more strongly with micropores than H_2_, giving rise to considerable adsorption. In contrast, subcritical CO_2_ with a large quadrupole moment can strongly interact with the micropores (*33*), leading to a more adsorption than CH_4_. The fractional fillings of pores by adsorbed CO_2_ and CH_4_ were approximately 0.3 and 0.1, respectively (Fig. 2D). Consequently, the selectivity of H_2_ against CO_2_ or CH_4_ using their mixed gases is reasonably smaller, due to the interaction of H_2_ with adsorbed CO_2_ or CH_4_ as discussed later on for H_2_/CH_4_ mixed gas separation.

### Permeability

**Fig. 3.**
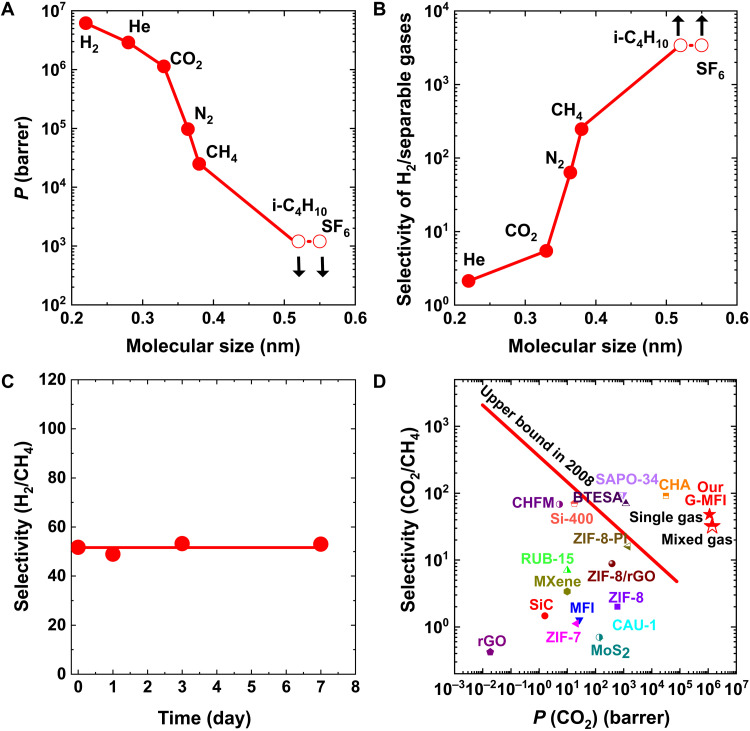
Gas separation performance of G-MFI membrane. (**A**) Variation in single-gas permeability with molecular size for the gases H_2_, He, CO_2_, N_2_, CH_4_, i-C_4_H_10_, and SF_6_. The MIN-2 molecular sizes of H_2_ and He are used. (**B**) Selectivity of H_2_ plotted against molecular size. Separable gases include He, CO_2_, N_2_, CH_4_, i-C_4_H_10_, and SF_6_. (**C**) Selectivity change with time for the separation of the H_2_/CH_4_ equimolar mixture. (**D**) Robeson plot for CO_2_/CH_4_. For the G-MFI membrane, both single and mixed gas CO_2_/CH_4_ selectivity is indicated for comparison. The red line shows the Robeson plot for polymeric membranes (*44*). Details of the Robeson plots with references are presented in table S1. The arrows in Fig. 2 (A and B) show that the permeability and selectivity for i-C_4_H_10_ and SF_6_ (open symbols) are maximum and minimum values, respectively.

The G-MFI membrane separation ability was investigated by gas permeability measurements with H_2_, He, CO_2_, N_2_, CH_4_, i-C_4_H_10_, and SF_6_, whose molecular size ranges from 0.26 nm for He to 0.55 nm for SF_6_. As shown in Fig. 3A, the permeability decreased with molecular size from H_2_ to CH_4_. H_2_ has a higher permeability than He because the molecular size of diatomic H_2_ [second minimum dimension (MIN-2): 0.22 nm] is smaller than that of He (MIN-2: 0.28 nm) (*34*). The smaller molecular mass of H_2_ compared to He also contributed to its higher permeability.

Furthermore, the selectivity of H_2_ against He, CO_2_, N_2_, CH_4_, i-C_4_H_10_, and SF_6_ for the G-MFI membranes was measured, and the corresponding Robeson plot is shown in Fig. 1A. The selectivity increased with increasing molecular size from H_2_/He to H_2_/CH_4_ (Fig. 3B). The H_2_/CH_4_ selectivity reached 245. For i-C_4_H_10_ and H_2_/SF_6_, the permeability was too low to be confidently measured; therefore, only an indication of maximum permeability and minimum selectivity is shown in Fig. 3, A and B. The G-MFI membrane has a significantly higher selectivity for H_2_ than the MFI membrane without wrapping (fig. S7). The MFI membrane contains cracks, resulting in lower H_2_ selectivity and higher permeability for other gases. Thus, the interfacial micropore spaces between graphene and MFI crystal faces impart outstanding separation ability to the G-MFI membrane. An equimolar mixture of H_2_/CH_4_ could be efficiently separated with a selectivity of 50, and the system was stable for more than 7 days (Fig. 3C). Noticeably, the mixed-gas selectivity was lower than that of the single-gas selectivity because of the hindrance effect of adsorbed CH_4_ molecules in the pores (Fig. 2D and movie S1). The decrease in H_2_/CH_4_ selectivity for a mixed gas is a result of an increase in CH_4_ permeability from 2.3 × 10^4^ barrers for single gas to 8.9 × 10^4^ barrers for a mixed gas.

Figure 3D shows the Robeson plot for CO_2_/CH_4_ separation. The G-MFI membrane exhibits a high selectivity for CO_2_ at a high permeability of 1.1 × 10^6^ barrers and a CO_2_/CH_4_ selectivity of 50 that is comparable to the CO_2_/CH_4_ separation using SAPO membranes (*35*). The CO_2_ permeability was slightly higher for the mixed gas than for the single gas. The CH_4_ mixed gas permeability of 4.9 × 10^4^ barrers was higher than the single gas permeability of 2.2 × 10^4^ barrers. Therefore, the CO_2_/CH_4_ selectivity decreased to 30 in the case of mixed gas steady permeability (fig. S8). The observed permeability behavior stems from the enhanced adsorption effect of the preadsorbed molecules (*36*). Both CO_2_ and CH_4_ were adsorbed on the pore walls of intergranular connection areas akin to the interfacial spaces, enhancing the adsorption by effectively narrowing the pores for further adsorption. As CO_2_ is more preadsorbed than CH_4_, CH_4_ can obtain an explicit merit for permeation compared with CO_2_.

### Permeability mechanisms

**Fig. 4.**
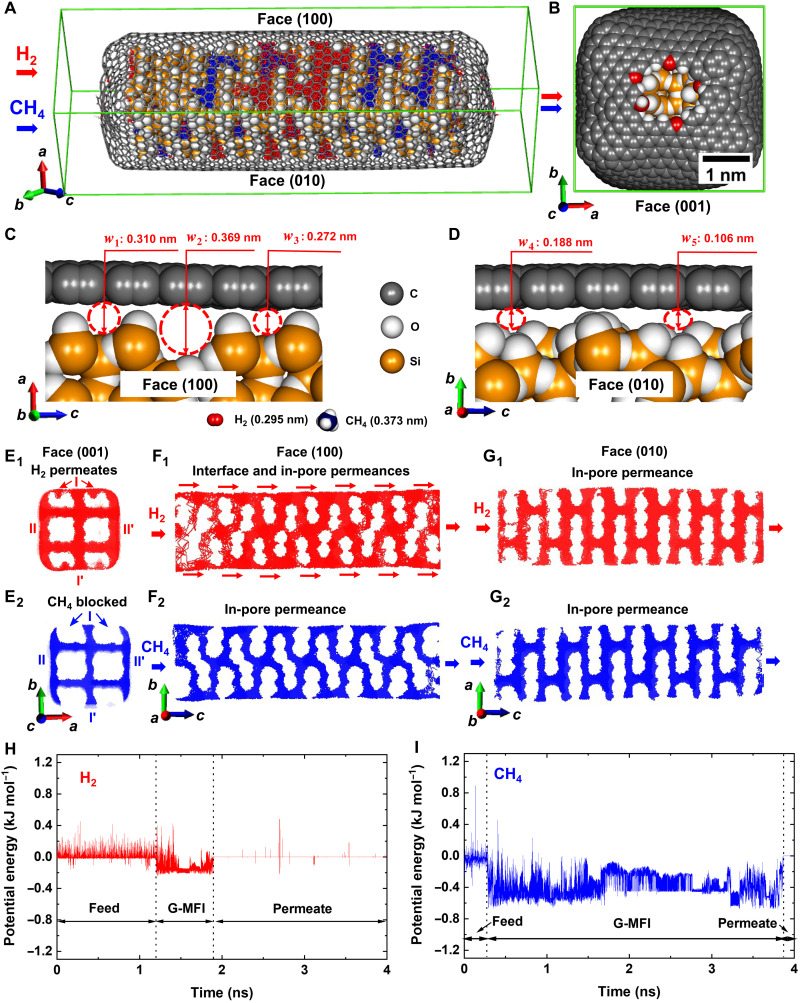
Permeability mechanism through the G-MFI crystal rod membrane model. (**A**) Permeation trajectories of H_2_ in red color and CH_4_ in blue color along the (100) and (010) MFI crystal faces of G-MFI crystal rod model. (**B**) Nanowindow on graphene contacted with the (001) MFI crystal face; surface oxygen atoms are in red. (**C**) Interface between graphene and (100) MFI crystal face. (**D**) Interface between graphene and (010) MFI crystal face. (**E**_1_ and **E**_2_) Trajectories of H_2_ and CH_4_ projected (001) face. The trajectories at the sides are denoted as I, I′, II, and II′, representing permeation at the graphene-MFI interfacial spaces. (**F**_1_ and **F**_2_) Trajectories projected to (100) face. Red arrows show permeation of H_2_ at the graphene-MFI interfacial spaces. Blue trajectory of CH_4_ shows that CH_4_ permeates through the pores, but not through the graphene-MFI interfacial spaces. (**G**_1_ and **G**_2_) Trajectories projected to (010) face, showing that H_2_ and CH_4_ permeate through the pores of MFI crystal. (**H** and **I**) Time courses of the potential energy of H_2_ and CH_4_.

MD simulations were further used to investigate the H_2_ and CH_4_ separation mechanisms (Fig. 4). MFI crystal rod models (fig. S9) of 7.885 and 43.368 nm in length along the *c* axis were used. As shown in Fig. 4A, the crystals were completely wrapped with single graphene, and nanowindows of ~1 nm in diameter were located at the left- and right-side centers, in contact with the left and the right (001) faces of the MFI crystal, as the inlet and outlet of the gases (Fig. 4B). The (010) face is predominant (fig. S2), whereas the (100) is the second predominant. Moreover, MFI crystals have straight channels (0.56 nm by 0.53 nm) along the *b* axis and sinusoidal channels (0.51 nm by 0.55 nm) along the *a* axis. These channels are terminated at the (010) and (100) crystal faces. Considering these observations, the adopted MFI crystal model showed (100) and (010) as the predominant crystal faces along the *c* axis (fig. S9). The gas permeates from the left nanowindow to the right nanowindow through the 2D interfacial space between graphene and the (010) and (100) faces of the MFI crystal rod, as indicated by the arrows in Fig. 4A. The effect of the length of the MFI crystal rod, interfacial surface area of the MFI crystal, and effective width (*w*_eff_) of the 2D interfacial space on H_2_ and CH_4_ permeation were examined. The examined G-MFI crystal rod model was at the equilibrium interface (*w*_eff_ = 0.03 nm) between graphene and MFI crystal faces, whereas G-MFI models of *w*_eff_ = 0.30 nm and *w*_eff_ = 0.40 nm are shown in fig. S10 (A to D) for comparison. Interfacial and pore structures were significantly affected by MFI crystal surface morphology (Fig. 4, C and D). The MFI unit cell has three grooves at the (100) face, causing surface roughness and affecting interfacial pore spaces size and shape. Effective pore widths of *w*_1_ = 0.310 nm, *w*_2_ = 0.369 nm, and *w*_3_ = 0.272 nm were observed (Fig. 4C). The interfacial spaces of *w*_1_ and *w*_2_ are compatible to the sole hydrogen (0.295 nm), thus giving rise to H_2_ permeability. Conversely, the groove sites between graphene and the smooth (010) crystal face were too narrow (*w*_4_ = 0.188 nm and *w*_5_ = 0.106 nm) for H_2_ and CH_4_ (Fig. 4D). Therefore, the interfacial space size governs the permeation and separation of H_2_ and CH_4_.

H_2_ and CH_4_ permeation pathways through the interfacial spaces between graphene and the (001), (100), and (010) faces are indicated with arrows in Fig. 4E. H_2_ permeates through the pores of MFI and interfacial spaces I, I′, II, and II′, providing continuous projected trajectories of H_2_ along all interfacial spaces of I, I′, II, and II′ (Fig. 4E_1_); contrarily, CH_4_ (0.37 nm) only permeates through the pores and interfacial spaces of II and II′, characterized by a groove structure (Fig. 4E_2_). H_2_ and CH_4_ trajectories through the pores of MFI and interfacial spaces between graphene and the (100) faces show evidently different transport pathways for H_2_ and CH_4_ (Fig. 4, F_1_ and F_2_). The red arrows at the top and bottom interfaces along the (100) face constitute the bold and continuous permeation pathways of H_2_, in addition to intrapore permeation, leading to the observed rapid permeation and selectivity (Fig. 4F_1_). CH_4_ trajectory pathways show that CH_4_ cannot pass through the interface between graphene and MFI crystal but only through the pores (Fig. 4F_2_). CH_4_ permeation is therefore remarkably smaller than that of H_2_, enhancing the H_2_/CH_4_ selectivity. Last, both H_2_ and CH_4_ cannot permeate the interfacial space between graphene and the (010) face of MFI, because of the less uneven surface structures than the (100) face. In-pore permeation, which can be observed in trajectories projected to the *ac* plane, is thus the only possible path (Fig. 4, G_1_ and G_2_). H_2_ and CH_4_ permeation pathways projected onto the *bc* plane are consequently different from each other. H_2_ molecules permeate through both the interfacial spaces between graphene and the (100) face and the MFI pores, whereas CH_4_ permeates only through the MFI pores. Hence, H_2_ molecules can bypass at the interfacial spaces and permeate faster than CH_4_ molecules. The important role of the interfacial bypass in the permeation of H_2_ was confirmed by the trajectory length. The average trajectory length of H_2_ molecules passing through the G-MFI (1170 nm) was 420 nm shorter than the average trajectory length of CH_4_ (1590 nm).

The time course of H_2_ and CH_4_ potential energy from the feed to permeate through G-MFI is shown in Fig. 4 (H and I). The potential energy of noninteracted H_2_ or CH_4_ in the gas phase as feed and permeate was set as 0 kJ mol^−1^. H_2_ or CH_4_ attractively interacts with pores of MFI and/or interfacial spaces, and thus the permeation time of H_2_ or CH_4_ in G-MFI can be determined by the duration of the negative potential energy zone (−0.2 kJ mol^−1^ for H_2_ and −0.6 kJ mol^−1^ for CH_4_), although repulsive collisions cause positive spikes in the potential energy. The feed is characterized by higher positive energy fluctuations than the permeate molecules, owing to the higher density of the inlet gas.

The total permeation time can be determined from potential energy/time graphs. H_2_ total permeation time through the nanoscale model membrane (0.7 ns) is five times shorter than that of CH_4_ (3.6 ns). The high permeation selectivity of G-MFI for H_2_ for the adopted nanoscale model further broadens at a micrometer level. Therefore, the permeation of H_2_ molecules through the bypass in the G-MFI membrane film can give a selectivity of H_2_/CH_4_ nearly 100 for a single gas.

**Fig. 5.**
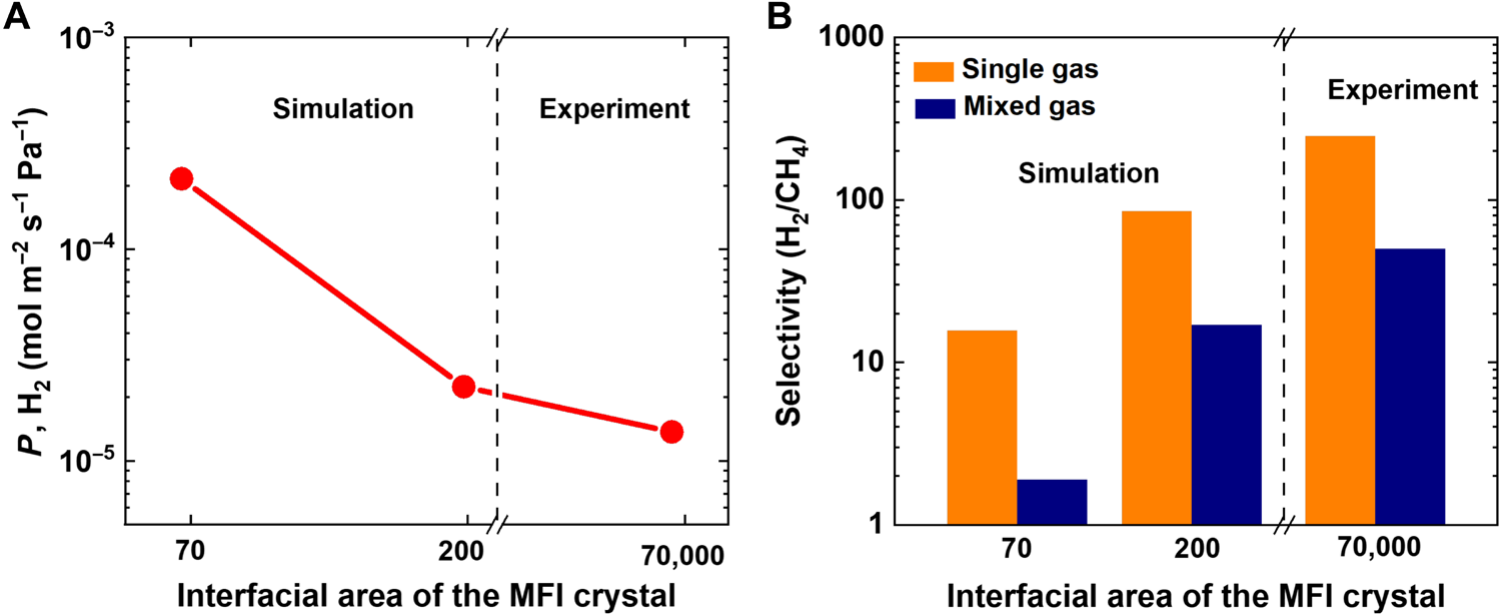
G-MFI permeability and selectivity. (**A**) H_2_ permeability plotted against the interfacial surface area of the MFI crystal, as determined by simulations and experiments. (**B**) H_2_/CH_4_ selectivity plotted against the interfacial surface area of the MFI crystal, as determined by simulations and experiments for single and mixed gases. Interfacial area is equal to the geometrical external area of MFI crystal for simulation and MFI particles for experiment.

The permeance of H_2_ decreases with the length of the model crystal (Fig. 5A). The interfacial space between graphene and MFI crystal face is the key for molecular sieving, as confirmed by simulation of the H_2_ and CH_4_ permeance through the MFI crystal (fig. S10, E and F). The H_2_ permeance through the G-MFI is smaller than that through the MFI model due to difference in the pore structure between MFI and G-MFI. The MFI has the pores of 0.55 nm, while the model structure of G-MFI has the interfacial 2D pores (Fig. 4, C and D) of 0.369 nm. The small 2D interfacial pores produced by graphene wrapping serve as the energy barriers for CH_4_, being responsible for molecular sieving (Fig. 4D). The larger interfacial area suggests that there are more energy barriers for H_2_ at the graphene-MFI interfacial spaces, affecting H_2_ permeability. The permeability reduction for CH_4_ was more prominent for the larger interfacial surface membranes. Accordingly, the selectivity for single gas and mixed gas increased with an increase in the interfacial area of the membrane (Fig. 5B). The synthetized G-MFI membrane can be regarded as an extreme case of model membranes. The G-MFI membrane is 150 μm thick; thus, the number of interfacial contacts between graphene and MFI crystals, which act as energy barriers, is considerable, resulting in higher experimental selectivity.

## DISCUSSION

G-MFI membranes efficiently separate H_2_ with high selectivity at an ultrahigh permeability of ~10^6^ barrers, due to both the narrow micropore spaces between graphene and zeolite crystal surface and the hierarchical pore structure. The activation energy for H_2_ permeation through the G-MFI membrane was 9.5 kJ mol^−1^ (fig. S11), supporting that the molecular sieving effect is due to narrow micropores, in agreement with the MD simulations. We examined the effect of zeolite crystal face effect on permeability. The graphene-wrapped BEA zeolite membrane had a significantly higher permeability than G-MFI, but almost no H_2_/CH_4_ selectivity (fig. S12). This is because the width of the interfacial space of the graphene-wrapped BEA zeolite is larger than the molecular size of H_2_ and CH_4_. Thus, the interfacial space should induce the observed molecular sieving effect of G-MFI for H_2_.

The G-MFI membrane is stable over 7 days, during which the selectivity of 50 for separation of an equimolar H_2_/CH_4_ mixture is maintained. The preparation of the crack-free G-MFI membrane via the illustrated facile route can be applied to highly efficient and economic H_2_ separation processes. The G-MFI membrane satisfies the requirements of the flux of 1.05 mol m^−2^ s^−1^ for efficient industrial separation (*37*); the flux of H_2_ through G-MFI membrane was measured to be 1.3 mol m^−2^ s^−1^. The graphene-wrapping methodology can be extended to the design of novel and highly performant separation membranes for any target gas, to ultimately promote a breakthrough toward green technologies.

## MATERIALS AND METHODS

### Wrapping of MFI zeolites with graphene and membrane preparation

Graphene wrapping of MFI zeolites was carried out in the colloidal state using GO and silicalite-1 (MFI zeolites) as raw materials. MFI crystals were synthesized by the sol-gel method, yielding a particle size of 300 nm (*38*). A solution consisting of tetrapropylammonium (TPA) hydroxide, sodium hydroxide, and distilled water at the stoichiometric ratio of 25 SiO_2_:2 (TPA)_2_O:1100 H_2_O:100 C_2_H_5_OH:0.1 Na_2_O was mixed at 298 K for 30 min (*38*). Tetraethyl orthosilicate was slowly added to the reaction mixture and stirred at 298 K for 24 hours. The reaction mixture was poured into a Teflon-lined autoclave and hydrothermally treated at 373 K for 24 hours. After hydrothermal treatment, white precipitation was obtained via filtration. The filtered powder was washed using boiling water and dried at 383 K. The MFI-type zeolite powder was calcined in air atmosphere at 773 K for 8 hours to remove organic structure-directing agents. GO was synthesized following the modified Hummers’ method (*39*). Madagascar graphite (5 g) was placed in a 1-liter beaker on a hot plate with a magnetic stirrer and thermometer. The magnetic stirring was set at 150 rpm. We slowly added 200 ml of concentrated H_2_SO_4_ and 22 ml of concentrated H_3_PO_4_, followed by 25 g of KMnO_4_, while maintaining a temperature of 308 to 313 K. The stirring speed was increased to 300 rpm and the reaction lasted for 5 hours. After the reaction, the beaker with the reaction mixture was placed in a plastic bowl containing ice, and 500 ml of water was slowly added, maintaining a temperature of <313 K. Then, 100 ml of H_2_O_2_ (10%) was slowly added. The GO was washed with 5% HCl five times to remove the remaining metal impurities. Last, the remaining acid was washed five times with water. The washed metals and acids were separated from GO by centrifugation. The GO was washed five times in water to exfoliate graphene sheets, and a diluted GO supernatant was collected.

The amount of GO was estimated to be sufficient for a single-layer wrapping of the external surface area of the MFI crystals (62 m^2^ g^−1^). Since the interactions between the MFI crystals and GO are weak in aqueous solution, we enhanced the interactions between the GO and MFI crystals by adding NH_4_Cl, which dissociates into ${\text{NH}}_{4}^{+}$ and Cl^−^ ions. ${\text{NH}}_{4}^{+}$ protonates the carboxyl functional groups, decreasing the surface energy or zeta potential of the GO layers and, thereby, facilitating the shrinking and wrapping process (*40*). An experimental flowchart for the wrapping of the MFI crystal with GO is shown in fig. S1.

We wrapped MFI zeolites with GO colloids (fig. S1A) by combining 50 mg of MFI zeolite crystals and 4.6 ml of a GO dispersion with a mass concentration of 0.04 weight % GO. We added 20 ml of 0.1 M NH_4_Cl to enhance interactions between the MFI and GO. After 24 hours, the GO enveloped the MFI crystals, leading to precipitation. We then froze the GO-wrapped MFI crystals with liquid nitrogen and removed frozen water with a freeze dryer to obtain the GO-wrapped MFI crystals (fig. S1B). The GO-wrapped MFI was thermally treated at a heating rate of 1 K min^−1^ in Ar at 623 K for 10 min to yield a G-MFI powder-like material, which was used for the fabrication of G-MFI membranes (fig. S1C).

The membrane was prepared by compression of G-MFI powder into a pellet using a compression die at a pressure of 550 MPa for 15 min. Compression yielded a 6-mm^2^ membrane (fig. S1, G to J). The size of the die used for making the membrane was 6 mm by 6 mm. We used 10 mg of G-MFI powder for membrane preparation using the die compression method. Using araldite adhesive, the membrane was mounted onto a polyacrylate plate holder with a 1-mm-diameter hole at the center.

### MD simulation

Classical MD simulations were conducted to elucidate the gas permeation mechanism through G-MFI membrane. We used large-scale atomic/molecular massively parallel simulator (LAMMPS) (*41*) to simulate the permeation of H_2_ and CH_4_ molecules. The H_2_ and CH_4_ molecules were set as single uncharged Lennard-Jones (LJ) centers, and the force field parameters are given in table S5. We conducted single and mixed gas permeance simulations with 1000 molecules in the simulation box. Equimolar mixture of H_2_ and CH_4_ was used for the mixed gas simulations. Periodic boundary conditions in the *a* and *b* directions were applied. The dimensions of the box in the *a* and *b* directions were set according to the size of the G-MFI model. The LJ 9-3 potential walls perpendicular to the *c* axis having small energy parameter were set at *c* = −300 nm and *c* = 50 nm. The G-MFI model was set at the origin in the coordinate system. The simulations were conducted at 298.15 K and the time step was 1 fs. The Nosé-Hoover thermostat was used to control the temperature. The total number of timesteps was 3 × 10^6^ (3 ns), which was enough time for H_2_ and CH_4_ permeance. We recorded the number of molecules permeated through the model G-MFI membrane every 50 MD steps.

We constructed a G-MFI membrane model based on an MFI crystal wrapped with graphene. We used the orthorhombic MFI crystal unit with lattice constants of *a* = 2.0090 nm, *b* = 1.9738 nm, and *c* = 1.3142 nm (*42*). We constructed the MFI crystal rod by extending the MFI crystal unit along the *c* axis. Si atoms in MFI were terminated with O atoms. The G-MFI model was built with packmol (*43*). The graphene layers were connected at the edges, and partial structural relaxation was performed for the edge parts using a discovery studio visualizer. Nanowindows of ~1 nm in diameter were opened at the center of both ends of the G-MFI rod model. After wrapping, the whole graphene structure was relaxed using the ReaxFF potential implemented in LAMMPS for the structural relaxation of the graphene structure.

MFI crystal rod models were used to calculate the permeance of the H_2_ and CH_4_ molecules. The effect of the crystal rod length on H_2_ and CH_4_ permeation through G-MFI was examined using 7.885- and 43.368-nm-long MFI crystals (fig. S7). The effective distance between graphene and MFI crystalline surface was set at 0.03 nm. An effect of the effective width on the permeation was examined using a 7.885-nm-long MFI crystal; the effective widths between graphene and the MFI crystal were 0.030, 0.30, and 0.40 nm (fig. S10).

We calculated the number of permeated molecules against time for each G-MFI rod model; the slope of the permeated molecules against time was found to be proportional to the molar flow in units of moles per second of the molecules through the G-MFI rod crystal. Dividing the molar flow in units of moles per second with the area of the G-MFI rod in units of square meters and transmembrane pressure difference in units of pascals gives the permeance expressed in units of moles per square meter per second per pascal. We used the areas of 10.60, 12.57, and 17.28 nm^2^ and the effective widths of 0.030, 0.30, and 0.40 nm, respectively, for calculation of the permeance. The transmembrane pressures were 8.4, 7.1, and 5.2 MPa for the effective widths of 0.030, 0.30, and 0.40 nm, respectively. High transmembrane pressures were used to determine the permeance with high statistical accuracy to obtain sufficient permeated molecules within the calculation time of a few hundreds of nanoseconds. The permeance was evaluated as expressed by the following equation

Permeance=N/(A·t·ΔP) (1)

where *N* denotes the number of permeated molecules, *A* is the area of the membrane, *t* is the time for molecules to permeate, and ∆*P* is the transmembrane pressure. We ran the simulation five times for each model and calculated the average permeances and selectivities.

### Explanation of effective width used in MD simulation

The effective width (*w*) is defined as follows

w=H–0.322 (nm) (2)

where *H* is the internuclear distance between the carbon atom in graphene and the oxygen atom on the outermost surface of the MFI crystal. A parameter of 0.322 nm corresponds to the sum of the van der Waals radii of a solid carbon atom in graphene and an oxygen atom in the MFI crystal.

## Supplementary Materials

### This PDF file includes:

Supplementary Text Figs. S1 to S12 Tables S1 to S5 References
