# Supplementary material for: Ultrapermeable 2D-channeled graphene-wrapped zeolite molecular sieving membranes for hydrogen separation
Source: Sci Adv. 2022 May 18;8(20):eabl3521. doi: 10.1126/sciadv.abl3521 (PMC9116883; doi:10.1126/sciadv.abl3521)
Supplement: Supplementary file 1 — Supplementary Text Figs. S1 to S12 Tables S1 to S5 References [file sciadv.abl3521_sm.pdf]

Science Advances
AAAS

Supplementary Materials for

Ultrapermeable 2D-channeled graphene-wrapped zeolite molecular sieving membranes for hydrogen separation

Radovan Kukobat, Motomu Sakai, Hideki Tanaka, Hayato Otsuka, Fernando Vallejos-Burgos, Christian Lastoskie, Masahiko Matsukata, Yukichi Sasaki, Kaname Yoshida, Takuya Hayashi, Katsumi Kaneko*

*Corresponding author. Email: kkaneko@shinshu-u.ac.jp

Published 18 May 2022, *Sci. Adv.* **8**, eabl3521 (2022)
DOI: 10.1126/sciadv.abl3521

The PDF file includes:

Supplementary Text
Figs. S1 to S12
Tables S1 to S5
Legend for movie S1
References

Other Supplementary Material for this manuscript includes the following:

Movie S1

Supplementary Text

Characterization

#1 Field Emission Scanning Electron Microscopy (FE-SEM)

G-MFI nanoparticles (Fig. 1B) were observed using a scanning electron microscope (SEM, JEOL, JSM 7000F). Additionally, we observed MFI crystals and the cross-sectional area of the G-MFI membrane (Fig. S1I, J). The G-MFI particles and the membrane were measured by simply placing the membrane material onto a carbon tape. The cross-sectional area of the G-MFI membrane was observed by tilting the sample specimen during observation.

#2 Thermogravimetric analysis

Thermal analyses for the membranes were carried out using a thermogravimetric analysis (TGA) apparatus (STA7200, Hitachi) in an air atmosphere. We observed the weight losses for the corresponding components of our G-MFI membrane (Fig. S1D). Weight losses arising from the decomposition of NH_4Cl and graphene were observed.

#3 Raman spectroscopy

The defects on the G-MFI membrane after thermal treatment in air at different temperatures were examined with Raman spectroscopy (Renishaw inVia, IAB 8303). Raman spectra were observed after thermal treatment of the G-MFI membrane in the temperature range of 300 – 723 K (Fig. S1E).

#4 Transmission Electron Microscopy (TEM)

We grounded the G-MFI membrane into powder, dispersed with ethanol, and placed on a Cu grid for observation by transmission electron microscopy (TEM, JEOL JEM 2100). In a similar way, we prepared the graphene and MFI for the TEM observations. The TEM examination of the G-MFI membrane was carried out to observe the graphene-zeolite crystal face contacts (Fig. 1C,D). The nanowindows on the graphene were observed (Fig. 1E, Fig. S2,3). We observed contact between graphene and the (010) face of the MFI zeolite crystal, and the same orientation of the MFI crystal was used in the MD simulation study.

#5 X-ray diffraction and small-angle X-ray scattering

An X-ray diffractometer (XRD) (Rigaku, CuKα, $\lambda = 0.15406$ nm) was used for diffraction measurement of the G-MFI membrane. XRD was measured before and after graphene wrapping (Fig. S5A) in transmission mode. We measured small-angle X-ray scattering (SAXS) for the G-MFI membrane and MFI membrane (Fig. S5B, C) using the synchrotron X-ray beam line BL8S3: wavelength (0.092 nm), camera length (0.45 m), and detector (R-AXIS, PILATUS, CCD) at the Aichi synchrotron radiation center. We calculated the radius of gyration using the Guinier approximation to discuss the porous structure of the G-MFI membrane.

#6 Gas adsorption

N_2 adsorption at 77 K for G-MFI in its powder and membrane forms was measured with an automatic volumetric gas adsorption apparatus (Quantacrome Autosorb iQ-MP) (Figs. 2A,B). H_2, CO_2, and CH_4 adsorption for the G-MFI membrane was measured using a

Microtrac MRB (BELSORP MAX) apparatus (Fig. 2C). The sample was degassed at 523 K for 3 h prior to the adsorption measurements.

#7 Gas permeance

Gas permeability measurements were carried out using a custom-made apparatus. Permeation tests for graphene-wrapped MFI membranes were carried out by using a pressure-driven method at 298 K. A membrane was fixed into a membrane module using O-rings. The pressure on the feed and permeate sides were set at 200 kPa and 100 kPa, respectively, and the permeate flow rate was measured using a bubble flow meter. In the case of separation tests for equimolar mixtures of H_2/CH_4 and CO_2/CH_4 the permeate flow of the mixture was measured by a bubble flow meter, and the composition of the permeate gas was determined using gas chromatography with a thermal conductivity detector (GC-TCD). The measurements were conducted after 1h of purging the equimolar H_2/CH_4 and CO_2/CH_4 mixtures at a feed pressure of 200 kPa, assuming that the H_2, CO_2, and CH_4 were pre-adsorbed on the G-MFI membrane. The steady permeabilities and selectivities were measured.

#8Diffusion coefficient estimation by solution diffusion transport

The diffusion coefficient is related to the permeance by the well-known permeation equation (*45*):

$$J = -\frac{DS\,\Delta p}{\delta}, \qquad (1)$$

where J is the molar flux of the permeate gas (mol m^{-2} s^{-1}), D is the diffusion coefficient (m^{2} s^{-1}), S is the solubility coefficient (mol m^{-3} Pa^{-1}), Δp is the transmembrane pressure (Pa), and δ is the thickness of the membrane (m).

The J to Δp ratio is equal to the permeance P (mol m^{-2} s^{-1} Pa^{-1}), and the diffusion coefficient is given by the following equation:

$$D = \frac{P\,\delta}{S} \qquad (2)$$

The permeance of H_2 through the G-MFI membrane was 1.3×10^{-5} mol m^{-2} s^{-1} Pa^{-1} and the thickness was 150 μm. The solubility coefficient of H_2 in the G-MFI membrane was determined using a molecular dynamics simulation model based on Henry's law:

$$S = \frac{c}{p} \qquad (3)$$

where c is the concentration of H_2 in the G-MFI membrane (mol m^{-3}) and p is the pressure of H_2 over the membrane of 8.5 MPa. The concentration of H_2 was calculated from the number of H_2 molecules in the upper part of the G-MFI membrane model using MD simulations. The average number of H_2 molecules in the upper part of the membrane model was 4.0 after 3 ns of simulation time. The volume of the upper part of the G-MFI

membrane model was 3.8×10^{-27} m^3, and a solubility coefficient of 2.0×10^{-4} mol m^{-3} Pa^{-1} was obtained using Equation (3). A diffusion coefficient of 9.5×10^{-2} cm^2 s^{-1} was calculated using Equation (2).

The diffusion coefficient for CH_4 obtained using the aformentioned method was 1.1×10^{-4} cm^2 s^{-1}. The ratio of the diffusion coefficients of H_2 and CH_4 was calculated as the diffusivity selectivity. The ratio of the solubility coefficients of H_2 and CH_4 was calculated as the solubility selectivity, as described in the main text.

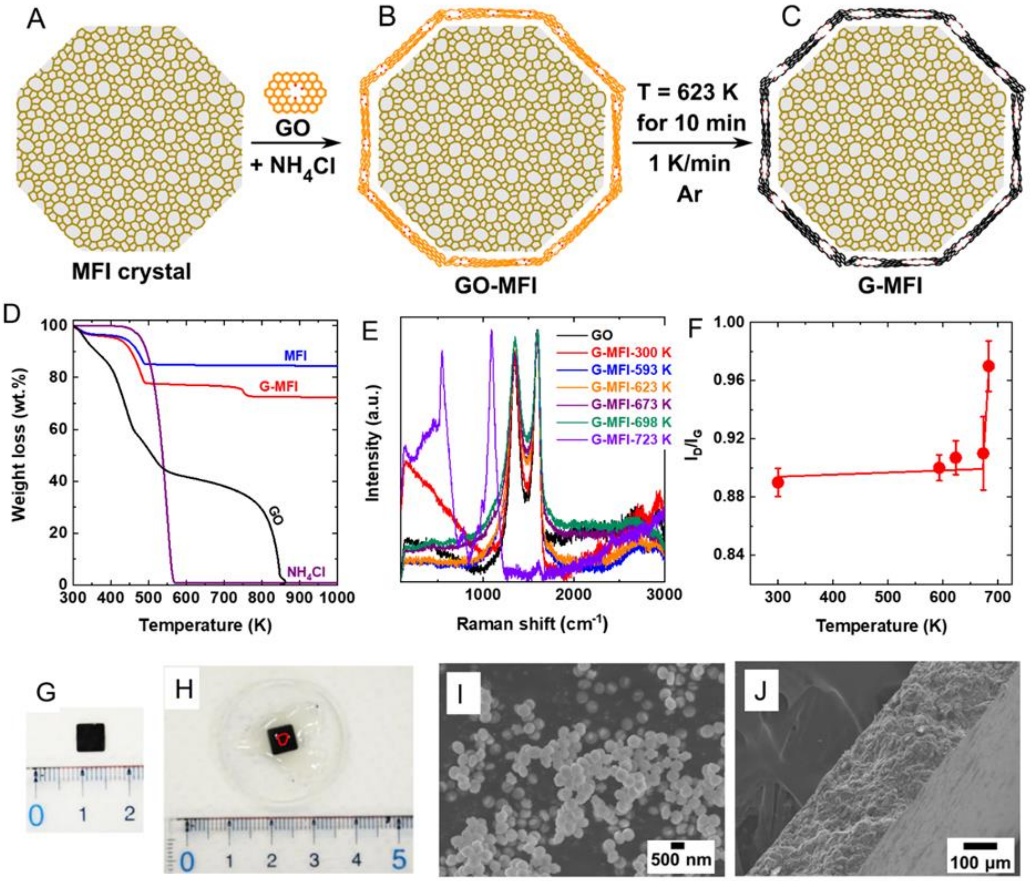

Fig. S1. Flowchart of the fabrication route for the graphene-wrapped MFI (G-MFI) membrane, starting from the wrapping of a MFI crystal with GO to the final product of a G-MFI crystal and its thermal stability characterization. (A) MFI crystal. (B) GO-wrapped MFI crystal. (C) G-MFI crystal. (D) TG curves for G-MFI crystals in air atmosphere at a heating rate of 5 K min^{-1}. (E) Raman spectra (λ = 532 nm) obtained for G-MFI crystals after thermal treatment in an air atmosphere at different temperatures. (F) The I_D/I_G ratio against temperature, showing that the I_D/I_G ratio almost does not increase with heating up to 673 K. (G) G-MFI membrane in pellet form. (H) G-MFI membrane mounted on a polyacrylate holder with araldite adhesive. The active area of 3.2×10^{-6} m^2 marked by the red circle was measured using ImageJ software. (I) SEM image of the MFI crystals, showing their spherical shape. (J) SEM image showing the cross-section of the membrane thickness.

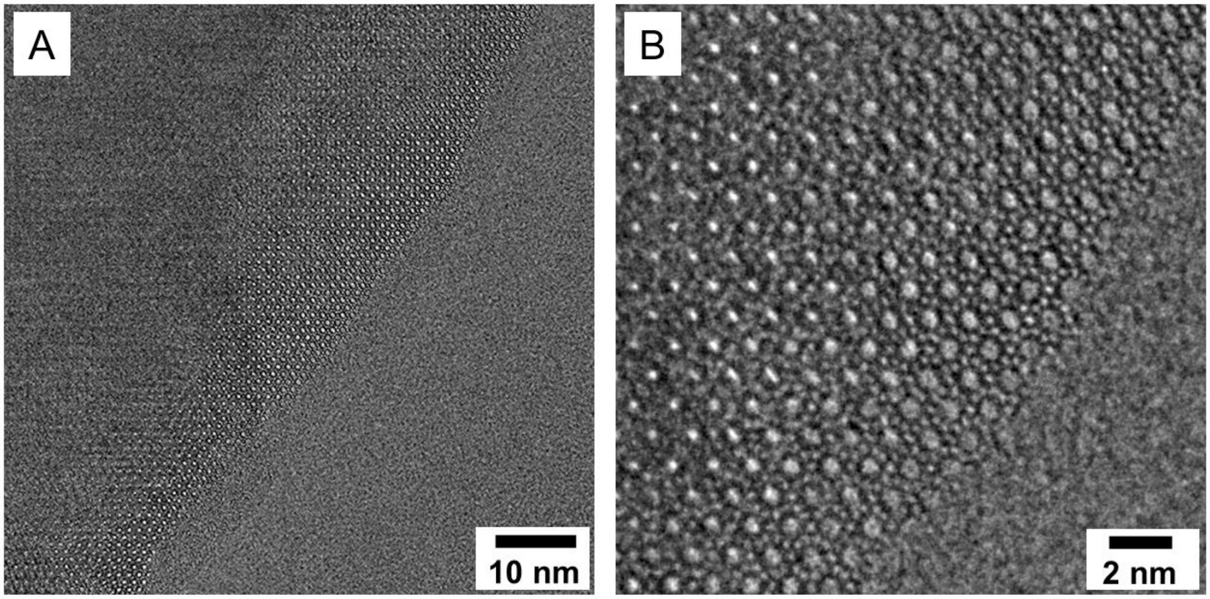

Fig. S2. TEM images of the (010) MFI crystal face. (A-B) Elliptical nanochannels of the MFI crystal at different magnifications.

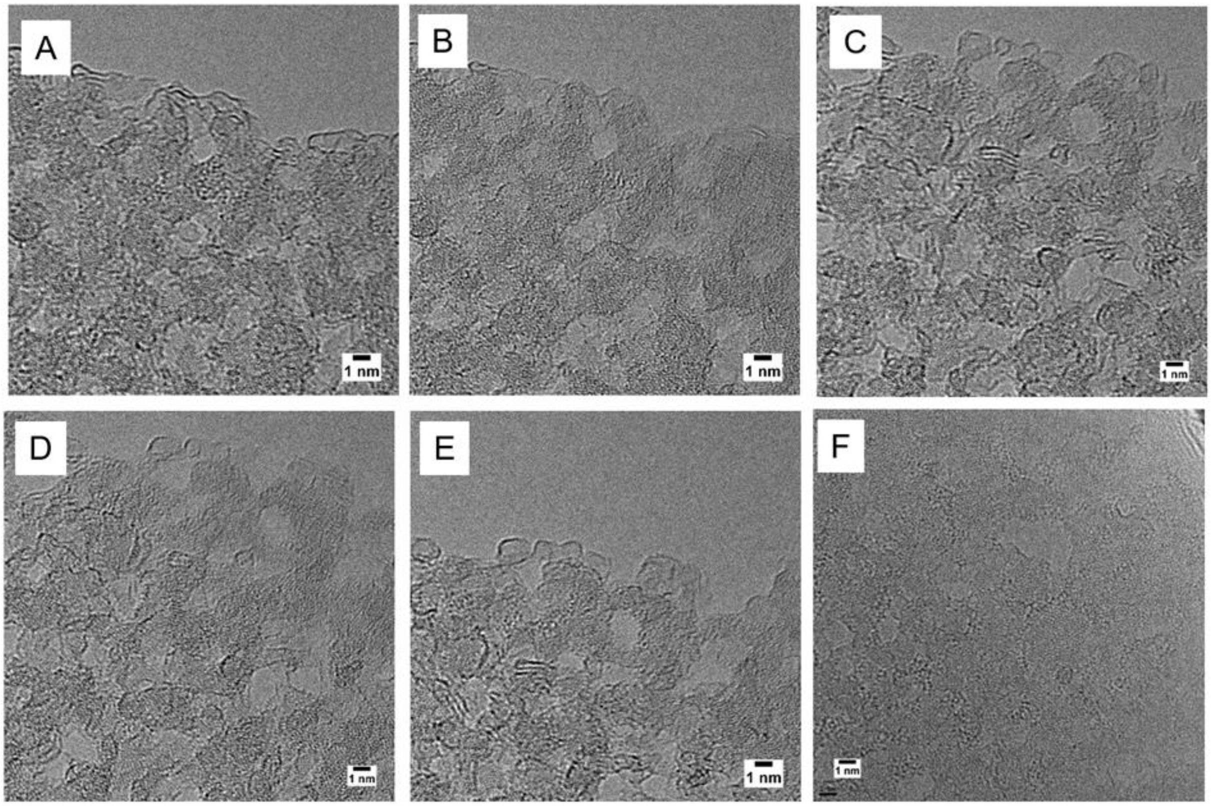

Fig. S3. TEM images of nanowindows on graphene for graphene-wrapped MFI (G-MFI) powder. (A-F) Nanowindows for different G-MFI powder samples observed at different positions.

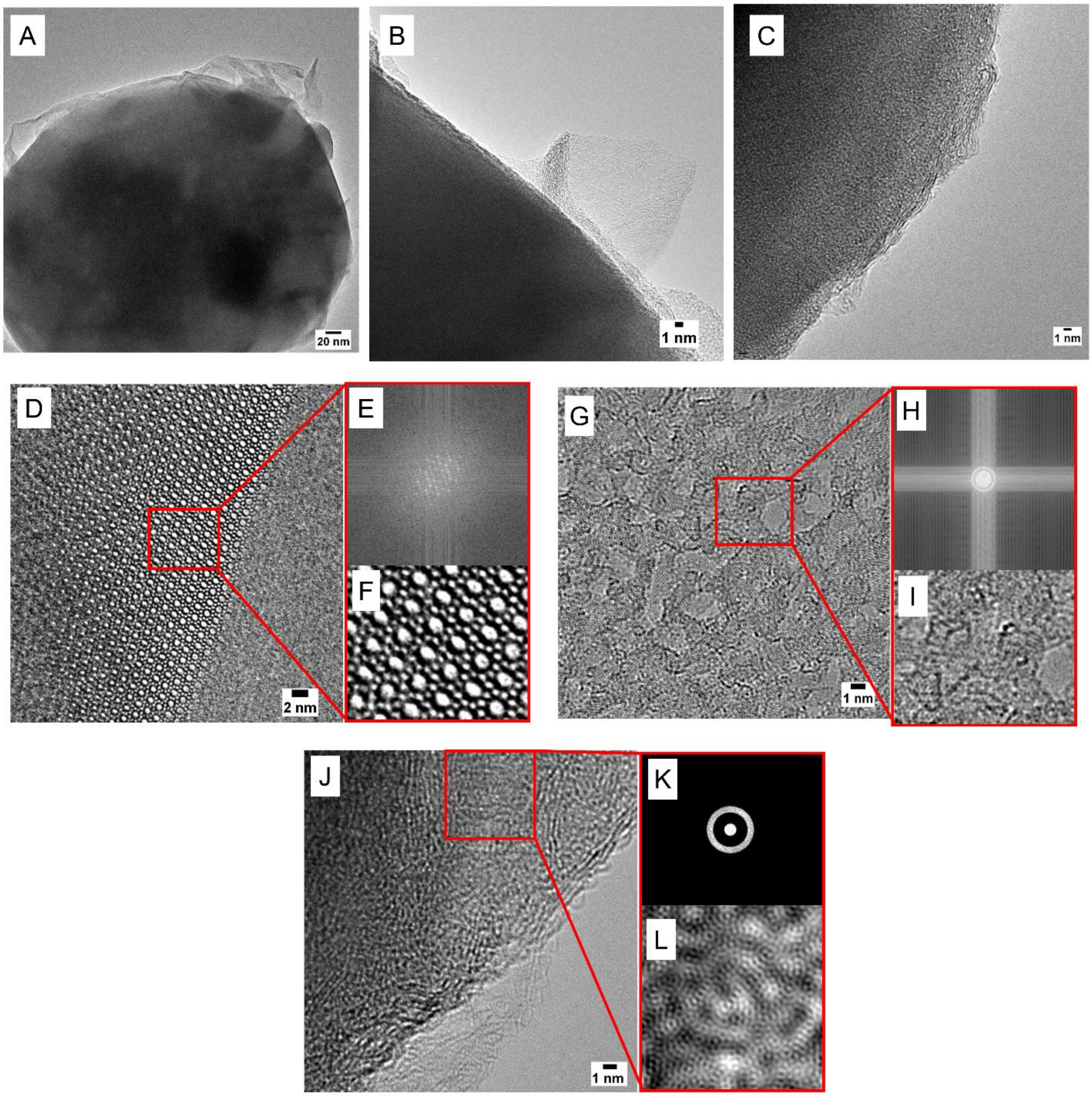

Fig. S4. TEM images of the G-MFI zeolites. (A) MFI zeolite wrapped with graphene. (B) Graphene layer attached to the surface of the MFI zeolite. (C) The graphene layers attached to the MFI crystal surface. (D) MFI crystal. (E) Fast Fourier Transform (FFT) and (F) inverse FFT for the SAED of the MFI crystal. (G) Graphene layer with nanowindows. (H) FFT and (I) IFFT for the SAED of the graphene. (J) Graphene layer on the MFI crystal surface. (K) FFT and (L) IFFT for the SEAD of the G-MFI.

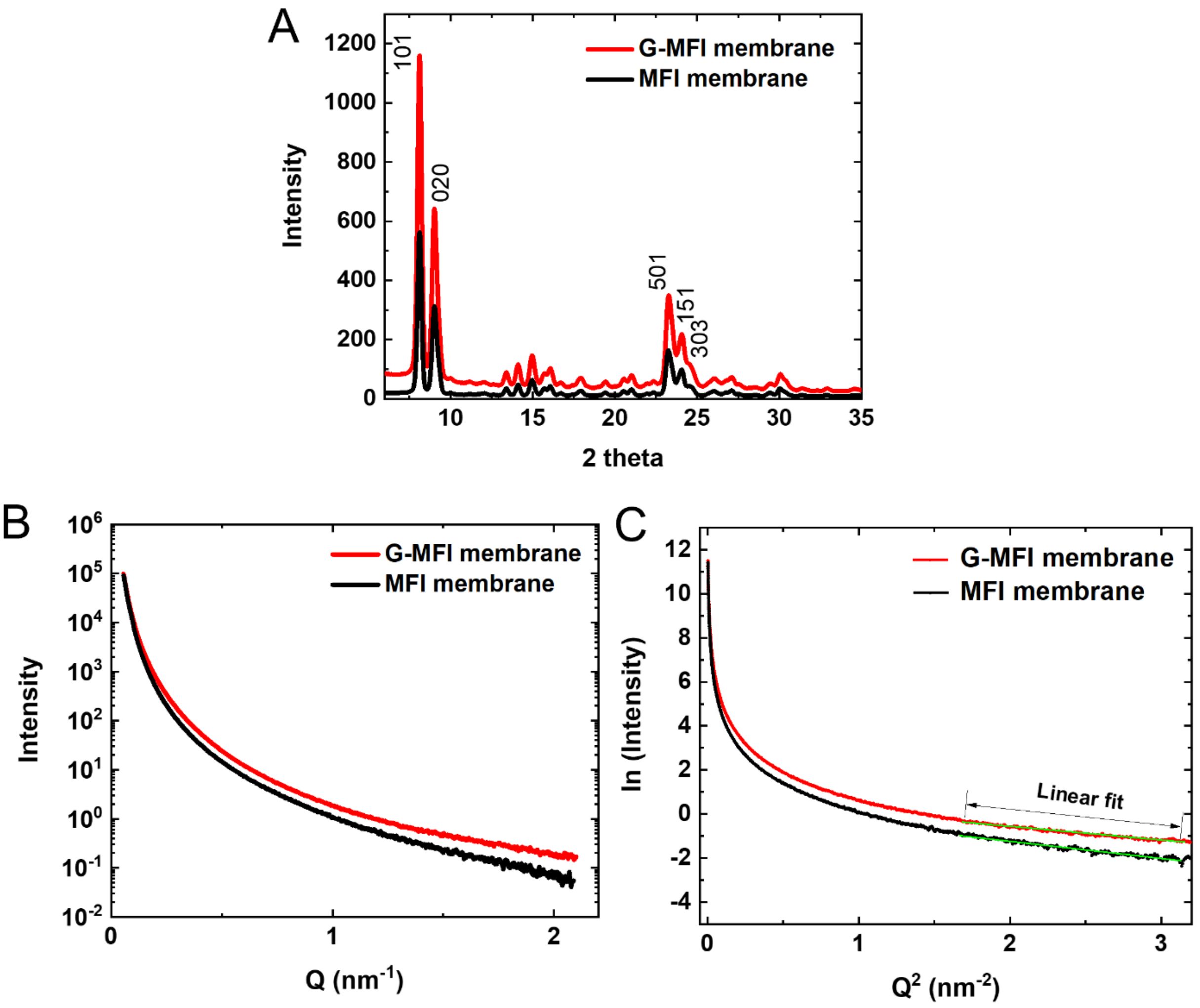

Fig. S5. XRD and SAXS of the G-MFI and MFI membranes. (A) XRD patterns for the graphene-wrapped MFI (G-MFI) and MFI membranes. (B) SAXS profiles for G-MFI and MFI membranes; intensity vs. scattering vector (Q). (C) Guinier plot. The radii of gyration for G-MFI and MFI membranes of 0.48 nm and 0.60 nm were determined from the linear region using the Guinier approximation (Table S4).

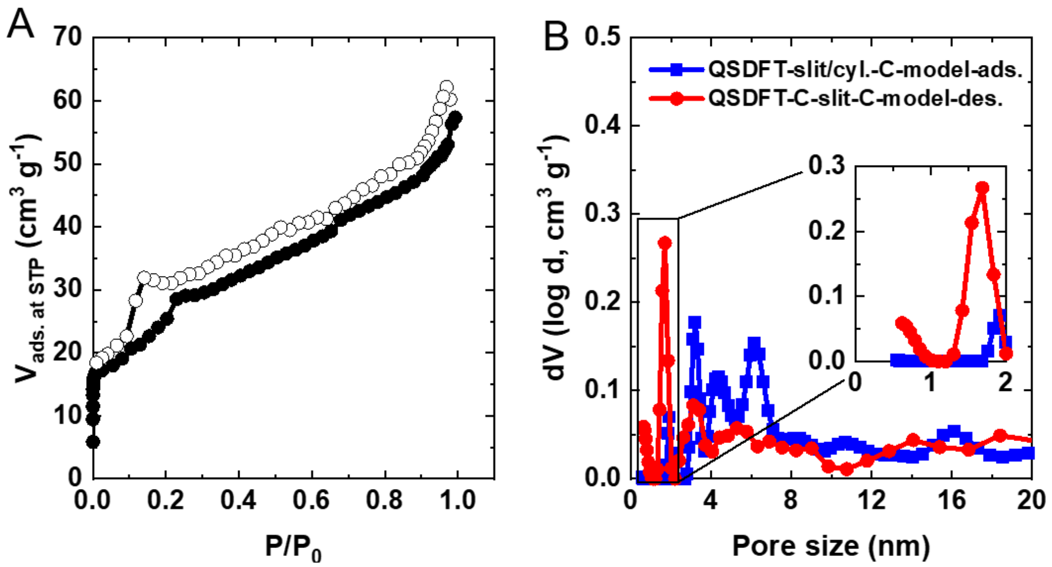

Fig. S6. The N_2 adsorption isotherm at 77 K and pore size distribution. (A) The N_2 adsorption isotherm obtained by subtraction of the adsorbed volume of N_2 of G-MFI with that of MFI membrane. (B) Pore size distribution of the G-MFI membrane obtained by QSDFT model. The QSDFT was applied to the adsorption branch for carbon slit/cylindrical pores (blue line) and to the desorption branch for carbon slit pores (red line).

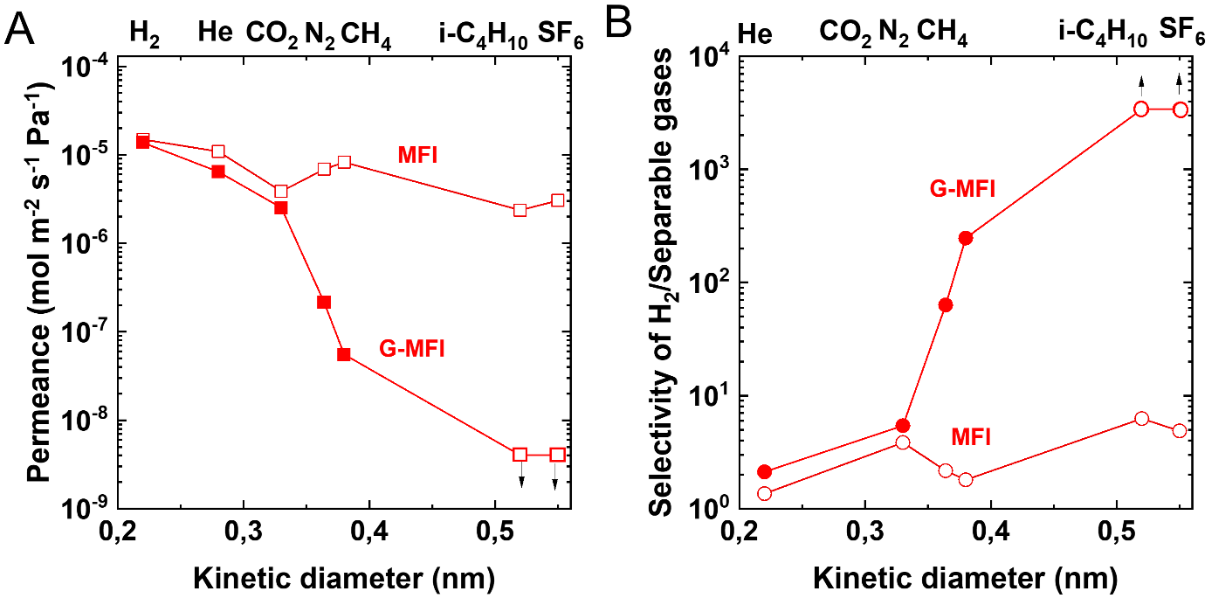

Fig. S7. Comparison of separation characteristics of the G-MFI and MFI membranes. (A) Permeability of a single gas against molecular size of a permeate gas. The lower side of the x-axis shows the kinetic diameter, and the upper side of the x-axis shows the corresponding gas molecule. (B) Selectivity of H_2 against the separable gases He, CO_2, N_2, CH_4, i-C_4H_{10}, and SF_6, as shown on the upper x-axis.

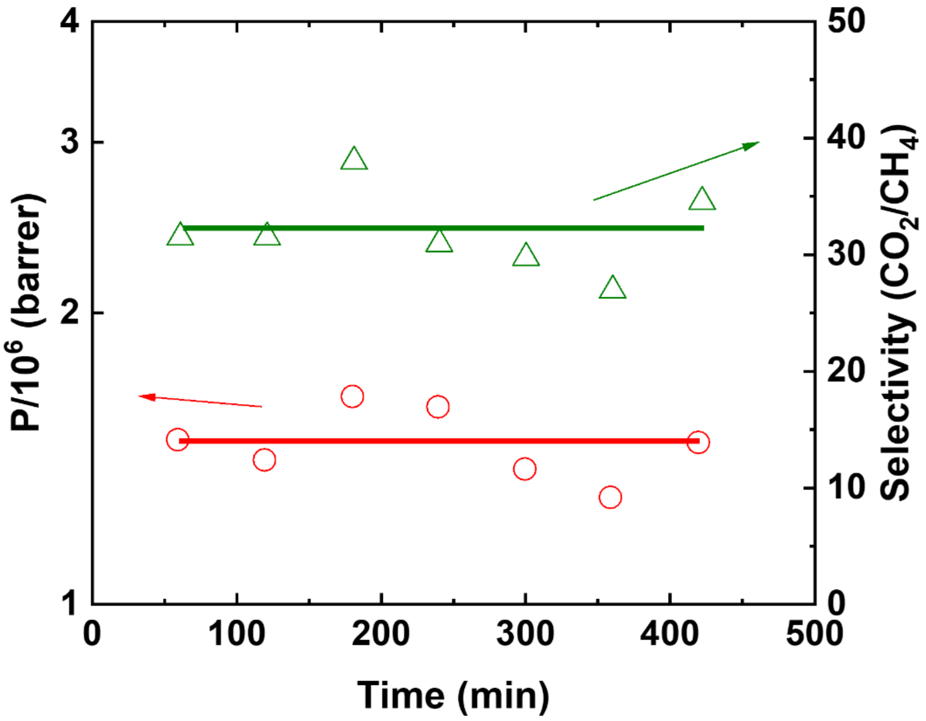

Fig. S8. Time-dependence of separation performance of G-MFI membrane. Permeability of CO_2 and selectivity of CO_2/CH_4 mixed gas against time.

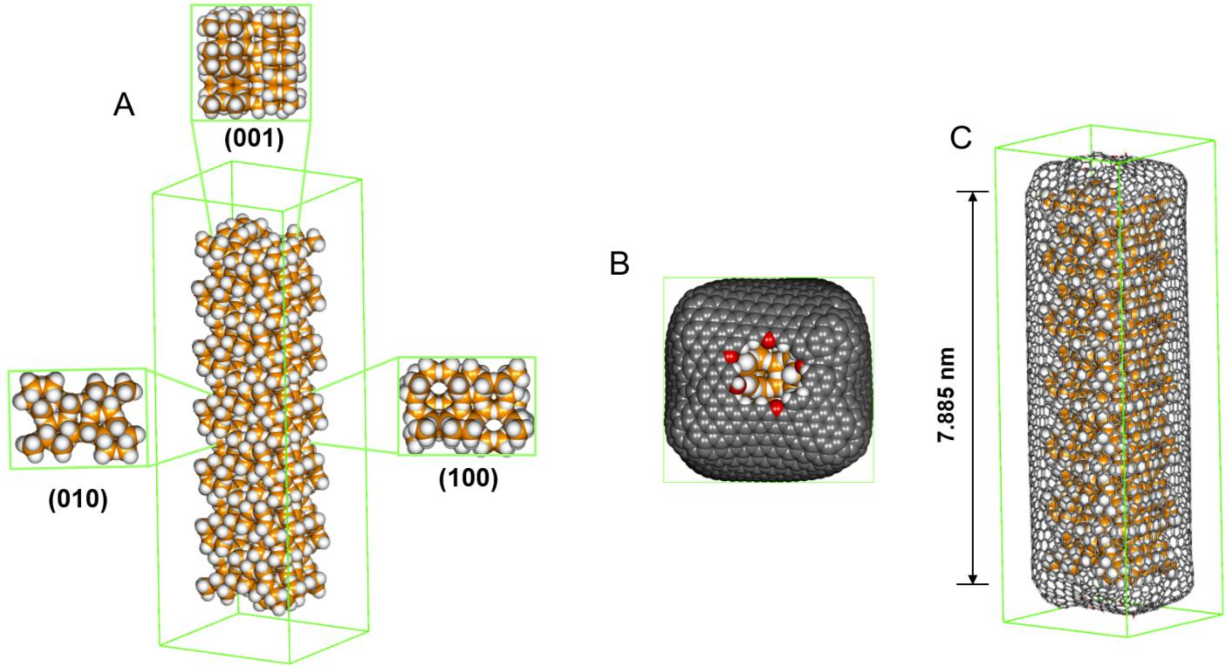

Fig. S9. Crystal rod models of the graphene-wrapped MFI (G-MFI) membranes for molecular dynamics simulations. (A) The crystal face structures of the MFI crystal; the (001) face is on the top, while the (010) and (100) faces are on the sides of the MFI crystal. The oxygen and silicon atoms in the MFI crystals are white and orange, respectively. (B) The G-MFI crystal viewed from the top; a ~1 nm diameter nanowindow on graphene located on the top of graphene. The oxygen atoms at the rim of the nanowindow are in red. Graphene is represented by the black color. (C) Side view of the MFI crystal wrapped with graphene whose length is 7.885 nm. The G-MFI model extended to 43.368 nm was also used as a model. For clarity, the atoms in the MFI crystal are drawn as silicon and oxygen atom spheres, graphene in figure (B) is drawn as carbon atom spheres, and the graphene in figure (C) is drawn with stick lines.

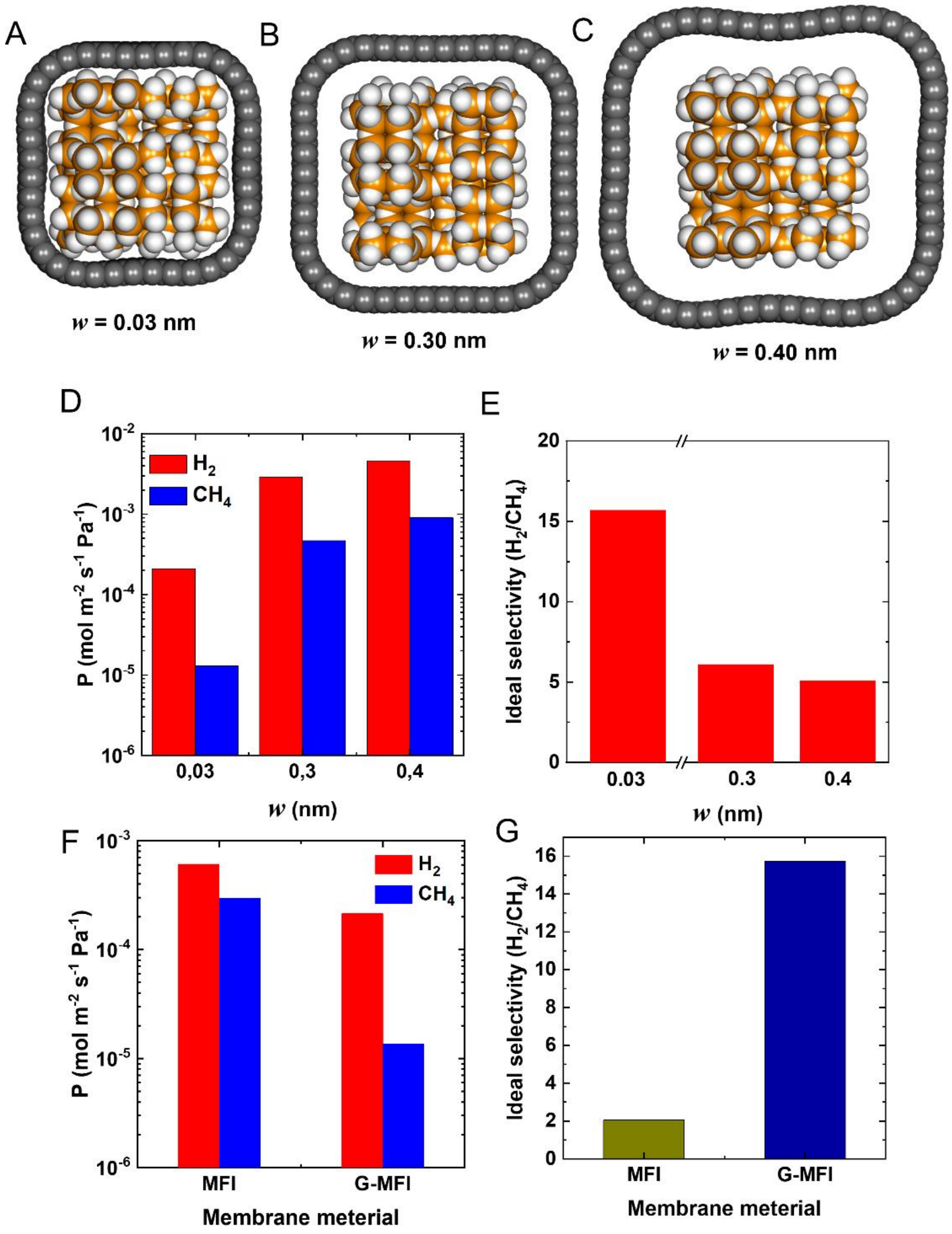

Fig. S10. Effect of the graphene-to-MFI surface effective width (*w*) on the permeance and ideal selectivity of G-MFI. (A) Cross section of the graphene-wrapped MFI (G-MFI) model with $w = 0.03$ nm. (B) Cross section of the G-MFI model of $w = 0.30$ nm. (C) Cross section of the G-MFI model for $w = 0.40$ nm. (D) Permeances of H_2 and CH_4 molecules against w. (E) Ideal selectivity against w. Comparison between permeance and ideal selectivity calculated by MD simulation using MFI and G-MFI membrane: (F) Permeance of H_2 and CH_4 through MFI and G-MFI, (G) Ideal selectivities of H_2/CH_4 by MFI and G-MFI. The MFI crystal road model of 7.885 nm was used for this simulation.

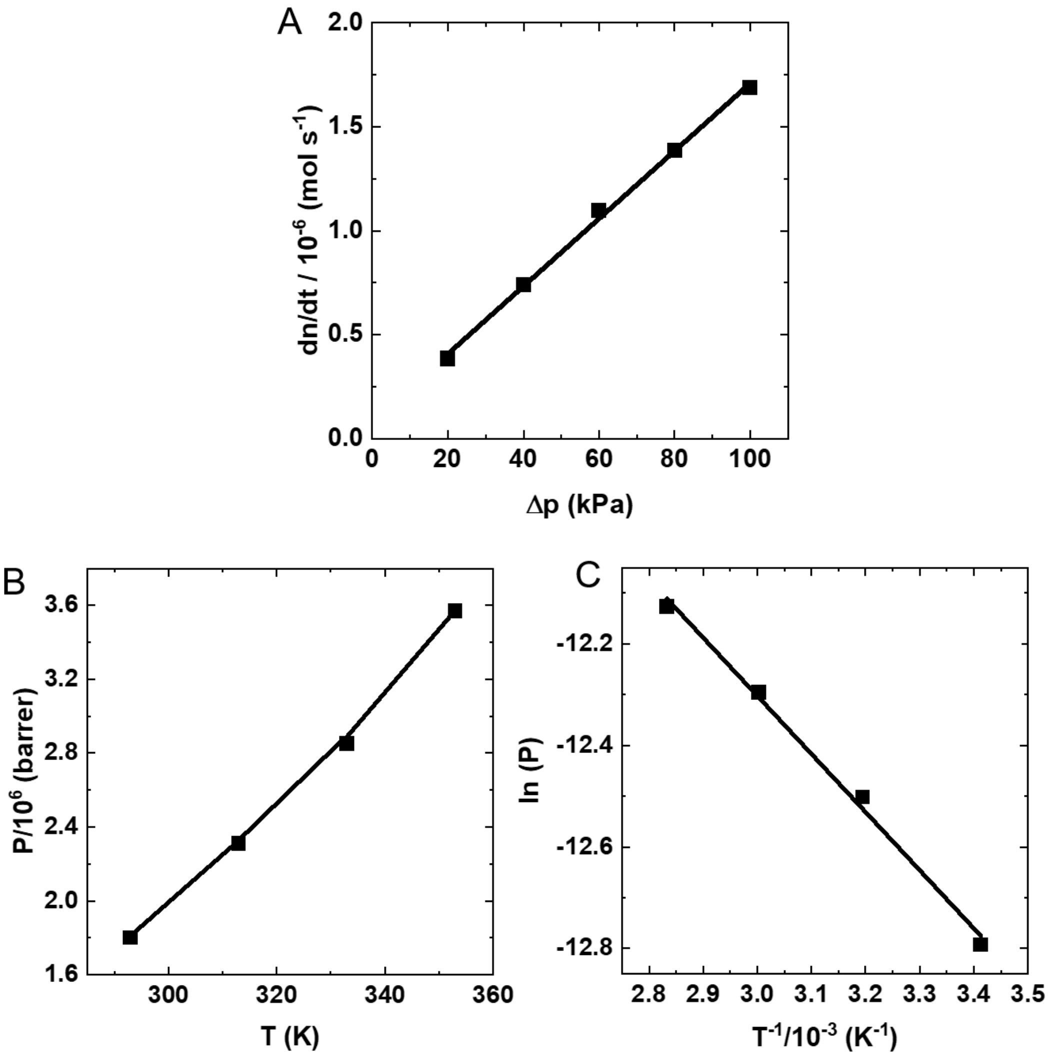

Fig. S11. Pressure and temperature dependence. (A) Molar flow of H_2 against transmembrane pressure (20 – 100 kPa) for the G-MFI membrane, suggesting the classical effusion mechanism in which the pore sizes of the G-MFI membrane are smaller than the mean free path of H_2. (B) Permeability of H_2 against temperature at a transmembrane pressure difference of 20 kPa. (C) Arrhenius plot for the temperature dependence of the H_2 permeance.

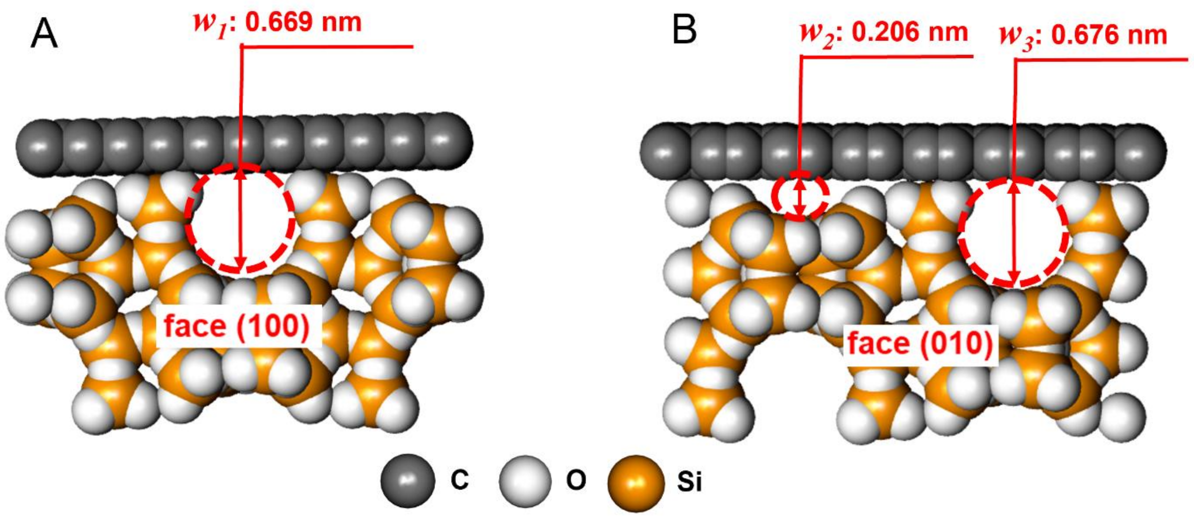

Fig. S12. Graphene-wrapped BEA zeolite interface. (A) Effective width w_1 between graphene and the (100) crystal face of the BEA crystal. (B) Effective widths w_2 and w_3 between graphene and the (010) crystal face of the BEA crystal. Experimental ideal selectivity of H_2/CH_4 by G-BEA was 1.95.

Table S1. Membrane characteristics. Permeabilities and ideal selectivities are shown as cited in the Robeson plots of Figures 1A and 3D.

Material	*t* (μm)	*T* (K)	H_2/ CO_2	H_2/ CH_4	CO_2/ CH_4	*P* (H_2)		*P* (CO_2)		Δp (atm)	Year[Ref.]
						barrer	mol m^{-2} s^{-1} Pa^{-1}	barrer	mol m^{-2} s^{-1} Pa^{-1}		
Si-400[1]	0.03	473	8	561	70	1.43×10^{2}	2.0×10^{-6}	1.80×10^{1}	2.0×10^{-7}	1.0	1998(*46*)
SiC	2	473	43	29	1.4	7.00×10^{1}	1.18×10^{-8}	1.60×10^{1}	2.8×10^{-10}	2.0	2007(*47*)
SAPO-34	5	295	-	-	95	-	-	9.55×10^{2}	6.4×10^{-8}	1.38	2008(*48*)
MFI	3	723	139	180	1.3	3.7×10^{3}	4.2×10^{-7}	2.70×10^{1}	3.0×10^{-9}	1.0	2009(*12*)
KUUST-1	60	298	4.5	7.8	-	2.15×10^{3}	1.2×10^{-6}	5.0×10^{4}	2.8×10^{-7}	1.0	2009(*49*)
ZIF-7	2	493	13	14.7	1.1	2.72×10^{2}	4.55×10^{-8}	2.10×10^{1}	3.5×10^{-9}	1.0	2010(*50*)
ZSM-5	9.5	723	25	-	-	6.32×10^{3}	2.2×10^{-7}	1.22×10^{3}	4.3×10^{-8}	1.0	2012(*13*)
rGO	0.018	298	279.4	117.6	0.42	6.21×10^{0}	1.0×10^{-7}	1.8×10^{-2}	3.6×10^{-10}	-	2013(*51*)
CAU-1	4	298	13.3	10.8	-	2.27×10^{3}	1.9×10^{-7}	1.79×10^{2}	1.5×10^{-8}	1.0	2013(*52*)
ZIF-8	6	303	5.8	11.1	1.9	3.56×10^{3}	19.9×10^{-8}	6.10×10^{2}	3.4×10^{-8}	0.2	2014(*53*)
ZIF-8/GO	20	523	22.4	198.3	8.8	8.36×10^{3}	1.4×10^{-7}	3.88×10^{2}	6.5×10^{-9}	1.0	2014(*54*)

MoS2	0.017	423	3.4	2.4	0.7	4.68×10^{2}	9.2×10^{-6}	1.39×10^{2}	2.7×10^{-6}	0.8	2015(*55*)
COF-MOF	60	298	12	6.2	-	1.0×10^{5}	5.5×10^{-7}	8.30×10^{3}	4.6×10^{-8}	1.0	2016(*56*)
2H MoS2	1	298	7.6	5.5	0.67	1.18×10^{3}	3.9×10^{-7}	1.46×10^{3}	4.9×10^{-8}	-	2016(*57*)
ZIF-8-PI[2] (30 %)	-	308	1.8	28	16	2.58×10^{3}	-	1.43×10^{3}	-	1.00	2016(*58*)
ZIF-90-6FDA-TP (50 %)	76	308	2.8	101	36	1.79×10^{2}	7.8×10^{-10}	6.30×10^{1}	2.7×10^{-10}	9.8	2017(*59*)
MXene	2	298	238	780	3.4	2.40×10^{3}	3.7×10^{-7}	1.0×10^{1}	1.7×10^{-9}	1.0	2018(*60*)
BTESA-A 9:1[3]	0.5	473	-	54	16.8	1.90×10^{3}	1.3×10^{-6}	5.52×10^{2}	3.7×10^{-7}	1.4	2020(*61*)
CHA	3	300	-	-	91	-	-	3.1×10^{4}	3.5×10^{-6}	1.0	2020(*62*)
Graphene	-	300	-	34	27	-	4.0×10^{-6}	-	3.2×10^{-6}	0.5-1.0	2021(*63*)
MOF-in-COF	1.00	298	33	38	-	3.20×10^{3}	1.1×10^{-6}	1.00×10^{2}	3.3×10^{-8}	1.00	2021(*64*)
RUB-15[4]	0.3	423	3	27	7	3.80×10^{1}	4.1×10^{-8}	1.00×10^{2}	1.2×10^{-8}	-	2021(*10*)
CHFM[5]	3.0	403	84	5706	68	4.75×10^{2}	5.3×10^{-8}	5.30×10^{1}	5.9×10^{-10}	1.0	2021(*65*)

G-MFI (Single gas)	150	300	5.5	247	48	5.80×10^{6}	1.30×10^{-5}	1.20×10^{6}	2.5×10^{-6}	1.0	This work
G-MFI (Mixed gas)	150	300	3.0	50	32	4.50×10^{6}	1.00×10^{-5}	1.40×10^{6}	3.3×10^{-6}	1.0	This work

1-silica calcinated at 400 °C abbreviated as Si-400. 2-The MMM membrane containing polyimide (PI), containing 30 % ZIF-8; 2-4,40-hexa-fluoroisopropylidine bisphthalic dianhydride-triptycene polyimide (6FDA-TP), containing 50 % ZIF-90. 3-bis(triethoxysilyl)acetylene (BTESA) and (3-amino- propyl) triethoxysilane (APTES) precursors at the ratio of 9:1. 4-sodalite precursor has six-membered rings of SiO_4 tetrahedra. 5-cellulose-based asymmetric carbon hollow fiber membrane.

Table S2. X-ray diffraction peak characteristics. The positions and FWHMs of X-ray diffraction peaks of G-MFI and MFI.

Membrane	Plane	2 θ (°)	FWHM (°)
G-MFI	101	8.11	0.29
	020	9.04	0.37
	501	23.38	0.52
	151	24.04	0.43
	303	24.58	0.54
MFI	101	8.11	0.29
	020	9.04	0.37
	501	23.37	0.48
	151	24.03	0.41
	303	24.60	0.47

Table S3. Porosity parameters of G-MFI and MFI membranes and their powders. We obtained the specific surface area (S_{BET}) from the BET plot (*66*) and the specific surface area ($S_{\alpha s}$), micropore volume ($V_{Micro.}$) and external surface area ($S_{ex.}$) by subtracting pore effect method from high resolution α_S-plot (*67*).

Membrane material	S_{BET} (m^2 g^{-1})	$S_{\alpha s}$ (m^2 g^{-1})	$V_{Micro.}$ (cm^3 g^{-1})	S_{ex} (m^2 g^{-1})
MFI (membrane)	340	557	0.10	66
G-MFI (membrane)	416	676	0.12	111
MFI (powder)	360	580	0.11	62
G-MFI (powder)	360	580	0.11	77

Table S4. Summary of gyration radii determined from the linear regions of the Guinier plots for small-angle X-ray scattering of the G-MFI and MFI membranes. The radius of gyration was determined for the thickness of the flat particle model using PRIMUS software.(*68*)

Range	G-MFI		MFI	
Q^2 (nm^{-2})	R_g (nm)	QR_g limit	R_g (nm)	QR_g limit
1.6 – 3.1	0.48 ± 0.00	0.60 – 0.84	0.60 ± 0.04	0.85 – 1.05

Table S5. Force field parameters. Atomic mases, charges, and Lennard-Jones(LJ) σ and ϵ parameters used in MD simulations of H_2 and CH_4 permeance through the G-MFI membrane.

Molecule	Mass (1.66×10^{-27} kg)	Charge (e)	LJ σ (nm)	LJ ϵ (K)	Reference
H_2	2.01588	0	0.2958	36.733	Ref.(*69*)
CH_4	16.04276	0	0.373	148	Ref.(*70*)
C (-C=)	12.011	0	0.336	28	Ref.(*18*)
C (C-O-H)	12.011	0.15	0.355	35.2	
O (C-O-H)	15.9994	-0.585	0.307	85.5	
H (C-O-H)	1.00794	0.435	0	0	
C (C-O-C)	12.011	0.25	0.38	35.2	
O (C-O-C)	15.9994	-0.5	0.3	59.5	
C (C-H)	12.011	-0.115	0.355	35.2	
H (C-H)	1.00794	0.115	0.242	15.1	
Si	28.085	1.413	2.97	31.98	Ref.(*71*)
O (Si-O-Si)	15.9994	-0.7065	3.011	52	

Movie S1. H_2/CH_4 mixed gas separation by G-MFI membrane; H_2 molecules-red color, CH_4 molecules-blue color. The H_2 molecules permeate faster than the CH_4 molecules through the G-MFI. The CH_4 molecules adsorb the the G-MFI and hinder the permeance of H_2 molecules. The selectivity of H_2/CH_4 from the mixed gas is smaller than ideal selectivity of H_2/CH_4 from the single gas, agreeing with the experimental results (Fig. 1A).

REFERENCES AND NOTES

1. N. Sazali, M. A. Mohamed, W. N. W. Salleh, Membranes for hydrogen separation: A significant review. *Int. J. Adv. Manuf. Technol.* **107**, 1859–1881 (2020).

2. Q. Qian, P. A. Asinger, M. J. Lee, G. Han, K. M. Rodriguez, S. Lin, F. M. Benedetti, A. X. Wu, W. S. Chi, Z. P. Smith, MOF-based membranes for gas separations. *Chem. Rev.* **120**, 8161–8266 (2020).

3. R. Magnus, A. Lyngfelt, Using steam reforming to produce hydrogen with carbon dioxide capture by chemical-looping combustion. International journal of hydrogen energy. *Int. J. Hydrog.* **31**, 1271–1283 (2006).

4. N. W. Ockwig, T. M. Nenoff, Membranes for hydrogen separation. *Chem. Rev.* **107**, 4078–4110 (2007).

5. R. Ullah, M. Khraisheh, R. J. Esteves, J. T. McLeskey, M. AlGhouti, M. Gad-el-Hak, H. V. Tafreshi, Energy efficiency of direct contact membrane distillation. *Desalination* **433**, 56–67 (2018).

6. C. Lastoskie, Caging carbon dioxide. *Science* **330**, 595–596 (2010).

7. D. S. Sholl, R. P. Lively, Seven chemical separations to change the world. *Nature* **532**, 435–437 (2016).

8. E. M. Flanigen, J. M. Bennett, R. W. Grose, J. P. Cohen, R. L. Patton, R. M. Kirchner, J. V. Smith, Silicalite, a new hydrophobic crystalline silica molecular sieve. *Nature* **271**, 512–516 (1978).

9. K. Varoon, X. Zhang, B. Elyassi, D. D. Brewer, M. Gettel, S. Kumar, A. Lee, S. Maheshwari, A. Mittal, C. Y. Sung, M. Cococcioni, L. F. Francis, A. V. McCormick, K. A. Mkhoyan, M. Tsapatsis, Dispersible exfoliated zeolite nanosheets and their application as a selective membrane. *Science* **334**, 72–75 (2011).

10. M. Dakhchoune, L. F. Villalobos, R. Semino, L. Liu, M. Rezaei, P. Schouwink, C. E. Avalos, P. Baade, V. Wood, Y. Han, M. Ceriotti, K. V. Agrawal Gas-sieving zeolitic membranes fabricated by condensation of precursor nanosheets. *Nat. Mater.* **20**, 362–369 (2021).

11. G. Bernardo, T. Araújo, T. da Silva Lopes, J. Sousa, A. Mendes, Recent advances in membrane technologies for hydrogen purification. *Int. J. Hydrogen Energy* **45**, 7313–7338 (2020).

12. Z. Tang, J. Dong, T. M. Nenoff, Internal surface modification of MFI-type zeolite membranes for high selectivity and high flux for hydrogen. *Langmuir* **25**, 4848–4852 (2009).

13. H. Wang, Y. S. Lin, Synthesis and modification of ZSM-5/silicalite bilayer membrane with improved hydrogen separation performance. *J. Memb. Sci.* **396**, 128–137 (2012).

14. T. S. Chung, L. Y. Jiang, Y. Li, S. Kulprathipanja, Mixed matrix membranes (MMMs) comprising organic polymers with dispersed inorganic fillers for gas separation. *Prog. Polym. Sci.* **32**, 483–507 (2007).

15. L. Cheng, G. Liu, J. Zhao, W. Jin, Two-dimensional-material membranes: Manipulating the transport pathway for molecular separation. *Accounts Mater. Res.* **2**, 114–128 (2021).

16. B. Zornoza, O. Esekhile, W. J. Koros, C. Téllez, J. Coronas, Hollow silicalite-1 sphere-polymer mixed matrix membranes for gas separation. *Sep. Purif. Technol.* **77**, 137–145 (2011).

17. H. Verweij, Inorganic membranes. *Curr. Opin. Chem. Eng.* **1**, 156–162 (2012).

18. F. V.-Burgos, F. X. Coudert, K. Kaneko, Air separation with graphene mediated by nanowindow-rim concerted motion. *Nat. Commun.* **9**, 1812 (2018).

19. D. Stevic, A. Furuse, F. V.-Burgos, R. Kukobat, K. Kaneko, Cu-phthalocyanine-mediated nanowindow production on single-wall carbon nanohorn. *Mol. Phys.* **119**, e1815883 (2021).

20. K. Murata, K. Hirahara, M. Yudasaka, S. Iijima, D. Kasuya, K. Kaneko, Nanowindow-induced molecular sieving effect in a single-wall carbon nanohorn. *J. Phys. Chem. B* **106**, 12668–12669 (2002).

21. Z. Lu, M. L. Dunn, Van der Waals adhesion of graphene membranes. *J. Appl. Phys.* **107**, 044301 (2010).

22. D. D. L. Chung, Review: Graphite. *J. Mater. Sci.* **37**, 1475–1489 (2002).

23. M. Sakai, T. Kaneko, Y. Sasaki, M. Sekigawa, M. Matsukata, Formation process of columnar grown (101)-oriented silicalite-1 membrane and its separation property for xylene isomer. *Crystals* **10**, 949 (2020).

24. J. Dong, K. Wegner, Y. S. Lin, Synthesis of submicron polycrystalline MFI zeolite films on porous ceramic supports. *J. Memb. Sci.* **148**, 233–241 (1998).

25. U. Beuscher, E. J. Kappert, J. G. Wijmans, Membrane research beyond materials science, *J. Membr. Sci.* **643**, 119902 (2022).

26. Y. Jeong, S. Kim, M. Lee, S. Hong, M. G. Jang, N. Choi, K. S. Hwang, H. Baik, J. K. Kim, A. Yip, J. Choi, A hybrid zeolite membrane-based breakthrough for simultaneous CO_2 capture and CH_4 upgrading from biogas. *ACS Appl. Mater. Interfaces* **14**, 2893–2907 (2022).

27. S. Wang, Y. Yoshikawa, Z. Wang, H. Tanaka, K. Kaneko, Highly oxidation-resistant graphene-based porous carbon as a metal catalyst support. *Carbon Trends* **3**, 100029 (2021).

28. I. Díaz, E. Kokkoli, O. Terasaki, M. Tsapatsis, Surface structure of zeolite (MFI) crystals. *Chem. Mater.* **16**, 5226–5232 (2004).

29. R. T. Ferrel, D. M. Himmelbau, Diffusion coefficients of hydrogen and helium in water. *AIChE* **13**, 702–798 (1967).

30. P. L. Llewellyn, J. P. Coulomb, Y. Grillet, J. Patarin, G. Andre, J. Rouquerol, Adsorption by MFI-type zeolites examined by isothermal microcalorimetry and neutron diffraction. 2. nitrogen and carbon monoxide. *Langmuir* **9**, 1852–1856 (1993).

31. U. Müller, K. K. Unger, Sorption studies on large ZSM-5 crystals: The influence of aluminium content, the type of exchangeable cations and the temperature on nitrogen hysteresis effects. *Charact. Porous Solids* **39**, 101–108 (1988).

32. N. Setoyama, M. Ruike, T. Kasu, T. Suzuki, K. Kaneko, Surface characterization of microporous solids with helium adsorption and small angle x-ray scattering. *Langmuir* **9**, 2612–2617 (1993).

33. I. Mitxelena, M. Piris, Molecular electric moments calculated by using natural orbital functional theory. *J. Chem. Phys.* **144**, 204108 (2016).

34. K. S. W. Sing, R. T. Williams, The use of molecular probes for the characterization of nanoporous adsorbents. *Part. Part. Syst. Charact.* **21**, 71–79 (2004).

35. M. A. Carreon, S. Li, J. L. Falconer, R. D. Noble, Alumina-supported SAPO-34 membranes for CO_2/CH_4 separation. *J. Am. Chem. Soc.* **130**, 5412–5413 (2008).

36. T. Ohba, T. Suzuki, K. Kaneko, Preformed monolayer-induced filling of molecules in micropores. *Chem. Phys. Lett.* **326**, 158–162 (2000).

37. J. W. Phair, R. Donelson, Developments and design of novel (non-palladium-based) metal membranes for hydrogen separation. *Ind. Eng. Chem. Res.* **45**, 5657–5674 (2006).

38. N. Ren, Z. J. Yang, X. C. Lv, J. Shi, Y. H. Zhang, Y. Tang, A seed surface crystallization approach for rapid synthesis of submicron ZSM-5 zeolite with controllable crystal size and morphology. *Microporous Mesoporous Mater.* **131**, 103–114 (2010).

39. D. C. Marcano, D. V. Kosynkin, J. M. Berlin, A. Sinitskii, Z. Sun, A. Slesarev, L. B. Alemany, W. Lu, J. M. Tour, Improved synthesis of graphene oxide. *ACS Nano* **4**, 4806–4814 (2010).

40. J. Rong, M. Ge, X. Fang, C. Zhou, Solution ionic strength engineering as a generic strategy to coat graphene oxide (GO) on various functional particles and its application in high-performance lithium-sulfur (Li-S) batteries. *Nano Lett.* **14**, 473–479 (2014).

41. A. P. Thompson, H. M. Aktulga, R. Berger, D. S. Bolintineanu, W. M. Brown, P. S. Crozier, P. J. in 't Veld, A. Kohlmeyer, S. G. Moore, T. D. Nguyen, R. Shan, M. J. Stevens, J. Tranchida, C. Trott, S. J. Plimpton, LAMMPS - A flexible simulation tool for particle-based materials modeling at the atomic, meso, and continuum scales. *Comp. Phys. Comm.* **271**, 10817 (2022).

42. Database of Zeolite Structures (2017). Structure Commission of the International Zeolite Association (IZA-SC), Retrieved from http://www.iza-structure.org/databases/.

43. L. Martínez, R. Andrade, E. G. Birgin, J. M. Martínez, PACKMOL: A graphical user interface for computational chemistry softwares. *J. Comput. Chem.* **30**, 2157–2164 (2009).

44. L. M. Robeson, The upper bound revisited. *J. Memb. Sci.* **320**, 390–400 (2008).

45. A. F. Ismail, K. C. Khulbe, T. Matsuura, *Gas Separation Membranes: Polymeric and Inorganic* (Springer International Publishing, 2015).

46. R. M. de Vos, H. Verwelj, High-selectivity, high-flux silica membranes for gas separation. *Science* **279**, 1710–1711 (1998).

47. B. Elyassi, M. Sahimi, T. T. Tsotsis, Silicon carbide membranes for gas separation applications. *J. Memb. Sci.* **288**, 290–297 (2007).

48. S. Li, J. L. Falconer, R. D. Noble, SAPO-34 membranes for CO_2/CH_4 separations: Effect of Si/Al ratio. *Microporous Mesoporous Mater.* **110**, 310–317 (2008).

49. H. Guo, G. Zhu, I. J. Hewitt, S. Qiu, “Twin copper source” growth of metal−organic framework membrane: $Cu_3(BTC)_2$ with high permeability and selectivity for recycling H_2. *J. Am. Chem. Soc.* **131**, 1646–1647 (2009).

50. Y. Li, F. Liang, H. Bux, W. Yang, J. Caro, Zeolitic imidazolate framework ZIF-7 based molecular sieve membrane for hydrogen separation. *J. Memb. Sci.* **354**, 48–54 (2010).

51. H. Li, Z. Song, X. Zhang, Y. Huang, S. Li, Y. Mao, H. J. Ploehn, Y. Bao, M. Yu, Ultrathin, Molecular-sieving graphene oxide membranes for selective hydrogen separation. *Science (80).* **342**, 95–98 (2013).

52. S. Zhou, X. Zou, F. Sun, H. Ren, J. Liu, F. Zhang, N. Zhao, G. Zhu, Development of hydrogen-selective CAU-1 MOF membranes for hydrogen purification by “dual-metal-source” approach. *Int. J. Hydrogen Energy* **38**, 5338–5347 (2013).

53. X. Zhang, Y. Liu, S. Li, L. Kong, H. Liu, Y. Li, W. Han, K. L. Yeung, W. Zhu, W. Yang, J. Qiu, New membrane architecture with high performance: ZIF-8 membrane supported on vertically aligned ZnO nanorods for gas permeation and separation. *Chem. Mater.* **26**, 1975–1981 (2014).

54. A. Huang, Q. Liu, N. Wang, Y. Zhu, Bicontinuous ZIF-8@GO membrane with enhanced hydrogen selectivity. *J. Am. Chem. Soc.* **136**, 14686–14689 (2014).

55. D. Wang, Z. Wang, L. Wang, L. Hu, J. Jin, Ultrathin membranes of single-layered MoS_2 nanosheets for high-permeance hydrogen separation. *Nanoscale* **7**, 17649–17652 (2015).

56. J. Fu, S. Das, G. Xing, T. Ben, V. Valtchev, S. Qiu, Fabrication of COF-MOF composite membranes and their highly selective separation of H2/CO_2. *J. Am. Chem. Soc.* **138**, 7673–7680 (2016).

57. A. Achari, S. Sahana, M. Eswaramoorthy, High performance MoS_2 membranes: Effects of thermally driven phase transition on CO_2 separation efficiency. *Energ. Environ. Sci.* **9**, 1224–1228 (2016).

58. Z. Wang, D. Wang, S. Zhang, L. Hu, J. Jin, Interfacial design of mixed matrix membranes for improved gas separation performance. *Adv. Mater.* **28**, 3399–3405 (2016).

59. Q. Zhang, S. Luo, J. R. Weidman, R. Guo, Preparation and gas separation performance of mixed-matrix membranes based on triptycene-containing polyimide and zeolite imidazole framework (ZIF-90). *Polymer (Guildf).* **131**, 209–216 (2017).

60. L. Ding, Y. Wei, L. Li, T. Zhang, H. Wang, J. Xue, L. X. Ding, S. Wang, J. Caro, Y. Gogotsi, MXene molecular sieving membranes for highly efficient gas separation. *Nat. Commun.* **9**, 155 (2018).

61. M. Guo, M. Kanezashi, H. Nagasawa, L. Yu, J. Ohshita, T. Tsuru, Amino-decorated organosilica membranes for highly permeable CO_2 capture. *J. Memb. Sci.* **611**, 118328 (2020).

62. S. Araki, Y. Okubo, K. Maekawa, S. Imasaka, H. Yamamoto, Preparation of a high-silica chabazite-type zeolite membrane with high CO_2 permeability using tetraethylammonium hydroxide. *J. Memb. Sci.* **613**, 118480 (2020).

63. S. Huang, S. Li, L. F. Villalobos, M. Dakhchoune, M. Micari, D. J. Babu, M. T. Vahdat, M. Mensi, E. Oveisi, K. V. Agrawal, Millisecond lattice gasification for high-density CO_2- and O_2-sieving nanopores in single-layer graphene. *Sci. Adv.* **7**, eabf0116 (2021).

4. H. Fan, M. Peng, I. Strauss, A. Mundstock, H. Meng, J. Caro, MOF-in-COF molecular sieving membrane for selective hydrogen separation. *Nat. Commun.* **12**, 38 (2021).

5. L. Lei, F. Pan, A. Lindbråthen, X. Zhang, M. Hillestad, Y. Nie, L. Bai, X. He, M. D. Guiver, Carbon hollow fiber membranes for a molecular sieve with precise-cutoff ultramicropores for superior hydrogen separation. *Nat. Commun.* **12**, 268 (2021)

66. S. Brunauer, P. H. Emmett, E. Teller, Adsorption of gases in multimolecular layers. *J. Am. Chem. Soc.* **60**, 309–319 (1938).

67. K. Kaneko, C. Ishii, M. Ruike, H. Kuwabara, Origin of superhigh surface area and microcrystalline graphitic structures of activated carbons. *Carbon N. Y.* **30**, 1075–1088 (1992).

68. P. V. Konarev, V. V. Volkov, A. V. Sokolova, M. H. J. Koch, D. I. Svergun, PRIMUS: A windows PC-based system for small-angle scattering data analysis. *J. Appl. Cryst.* **36**, 1277–1282 (2003).

69. K. S. Deeg, J. J. G.-Sevillano, R. B.-Pérez, J. B. Parra, C. O. Ania, M. Doblaré, S. Calero, Insights on the molecular mechanisms of hydrogen adsorption in zeolites. *J. Phys. Chem. C* **117**, 14374–14380 (2013).

70. M. G. Martin, J. I. Siepmann, Transferable potentials for phase equilibria. 1. United-atom description of *n*-alkanes. *J. Phys. Chem. B* **102**, 2569–2577 (1998).

71. B. Vujic, A. P. Lyubartsev, Transferable force-field for modelling of CO_2, N_2, O_2 and Ar in all silica and Na^+ exchanged zeolites. *Model. Simul. Mater. Sci. Eng.* **24**, 045002 (2016).
